# The causal mutation leading to sweetness in modern white lupin cultivars

**DOI:** 10.1126/sciadv.adg8866

**Published:** 2023-08-04

**Authors:** Davide Mancinotti, Katarzyna Czepiel, Jemma L. Taylor, Hajar Golshadi Galehshahi, Lillian A. Møller, Mikkel K. Jensen, Mohammed Saddik Motawia, Bárbara Hufnagel, Alexandre Soriano, Likawent Yeheyis, Louise Kjaerulff, Benjamin Péret, Dan Staerk, Toni Wendt, Matthew N. Nelson, Magdalena Kroc, Fernando Geu-Flores

**Affiliations:** ^1^ Section for Plant Biochemistry and Copenhagen Plant Science Centre, Department of Plant and Environmental Sciences, University of Copenhagen, Thorvaldsensvej 40, 1871 Frederiksberg, Denmark.; ^2^ Legume Genomics Team, Institute of Plant Genetics, Polish Academy of Sciences, Strzeszyńska 34, Poznań, Poland.; ^3^ Royal Botanic Gardens Kew, Wakehurst Place, Ardingly, West Sussex RH17 6TN, UK.; ^4^ Traitomic A/S, J.C. Jacobsens Gade 14, 1799 Copenhagen, Denmark.; ^5^ IPSiM, University of Montpellier, CNRS, INRAE, Institut Agro, Montpellier, France.; ^6^ Amhara Agricultural Research Institute, Bahir Dar, Ethiopia.; ^7^ Department of Drug Design and Pharmacology, University of Copenhagen, Universitetsparken 2, 2100 Copenhagen, Denmark.; ^8^ Agriculture and Food, Commonwealth Scientific and Industrial Research Organisation, Floreat, WA 6014, Australia.; ^9^ The UWA Institute of Agriculture, The University of Western Australia, 35 Stirling Highway, Perth, WA 6009, Australia.

## Abstract

Lupins are high-protein crops that are rapidly gaining interest as hardy alternatives to soybean; however, they accumulate antinutritional alkaloids of the quinolizidine type (QAs). Lupin domestication was enabled by the discovery of genetic loci conferring low QA levels (sweetness), but the precise identity of the underlying genes remains uncertain. We show that *pauper*, the most common sweet locus in white lupin, encodes an acetyltransferase (AT) unexpectedly involved in the early QA pathway. In *pauper* plants, a single-nucleotide polymorphism (SNP) strongly impairs AT activity, causing pathway blockage. We corroborate our hypothesis by replicating the *pauper* chemotype in narrow-leafed lupin via mutagenesis. Our work adds a new dimension to QA biosynthesis and establishes the identity of a lupin sweet gene for the first time, thus facilitating lupin breeding and enabling domestication of other QA-containing legumes.

## Teaser

A single-nucleotide mutation enabled the modern domestication of white lupin by blocking the production of toxic alkaloids.

## INTRODUCTION

In the last decade, European efforts to diversify the local production of protein crops have intensified. Among the drivers are the impending shift toward plant protein-rich diets (*1*) and the awareness of the environmental impact of soybean cultivation and import (*2*). Lupins (*Lupinus* spp.) are the only protein crops whose seed protein content (up to 44%) can rival that of soybean (*3*). Moreover, lupins are relatively more tolerant to several abiotic stresses than other legumes and have great potential for the recovery of poor soils (*3*). However, lupin seeds naturally accumulate bitter and toxic alkaloids of the quinolizidine type (QAs) (*4*). The modern domestication of lupins began in the 1930s with the identification of low QA (“sweet”) lines whose seeds could be consumed directly without debittering (*5*). Starting in the 1960s, the addition of agronomic traits such as loss of seed dispersal and early flowering enabled the expansion of sweet lupin cultivation (*6*). Now, the two most widely cultivated species are white lupin (*Lupinus albus*) and narrow-leafed lupin (*Lupinus angustifolius*). Their respective centers of diversity lie in the Mediterranean basin (*7*, *8*) and the cultivation of sweet varieties now extends to a range of European and African countries as well as Australia and Chile. In Ethiopia, the severe shortage of protein for animal feed has sparked recent efforts to develop sweet white lupin cultivars adapted to local climates (*9*).

**Fig. 1.**
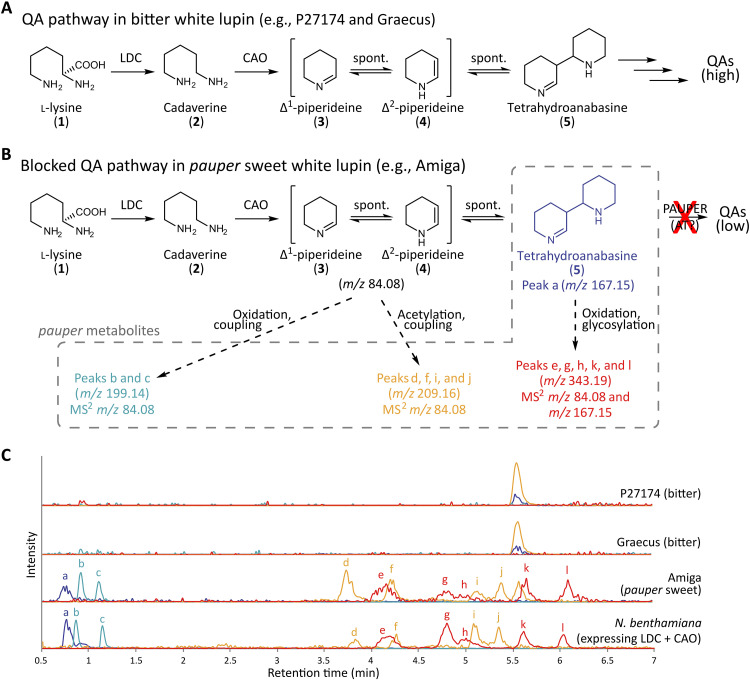
The distinctive chemotype of sweet white lupins of the *pauper* type. (**A**) Early QA biosynthesis pathway in bitter lupins according to the prevalent pathway hypothesis. spont.: spontaneous reaction. (**B**) Proposed QA pathway blockage accounting for sweetness in *pauper* white lupin. Distinctive metabolites accumulate in *pauper* plants (*pauper* metabolites), suggesting that the blockage occurs just downstream of the step catalyzed by CAO. Dashed arrows show the formation of *pauper* metabolites upon accumulation of the immediate product of CAO (Δ^1^-piperideine, **3**) and its spontaneously formed dimer (tetrahydroanabasine, **5**). The origin of the *pauper* metabolites was inferred from their MS^2^ spectra (fig. S1), particularly from the presence of fragments of *m*/*z* 84.08 (corresponding to Δ^1^-piperideine, **3**) and *m*/*z* 167.15 (corresponding to tetrahydroanabasine, **5**). (**C**) Representative LC-MS chromatograms of leaf extracts from two bitter (P27174, Graecus) and one *pauper* sweet (Amiga) white lupin lines (top three chromatograms) as well as an extract of *N. benthamiana* leaves transiently expressing LDC and CAO (bottom chromatogram). Accumulation of *pauper* metabolites is observed in the leaves of *N. benthamiana* upon transient coexpression of LDC and CAO. Traces are extracted ion chromatograms (EICs) of the most representative *pauper* metabolites (mean *m*/*z* ± 0.01). Signal intensities were adjusted to aid visualization (see Materials and Methods for scaling factors).

A few individual loci conferring sweetness exist; however, the precise identity of the underlying genes remains uncertain. QAs are known to be synthesized in green tissues and transported to the seed (*10*), but the QA pathway remains largely unknown (Fig. 1A). Of the hypothetical six to nine biosynthetic enzymes (*11*), only the first two are known: lysine decarboxylase (LDC) (*12*) and copper amine oxidase (CAO) (*13*) (Fig. 1A). Because the known sweet loci confer sweetness in both seeds and green tissues, uncovering the underlying genes will not only aid trait introgression but also help uncover the QA pathway. In white lupin (*L. albus* L.), the most widely exploited sweet locus is *pauper*, which lowers seed QA content down to 0.02 to 0.05% dry seed weight (*14*). The first molecular marker for *pauper* was published in 2008 and lay at a genetic distance of 1.8 cM (*15*). Recently, the sequencing of the white lupin genome allowed the mapping of *pauper* to a 958-kb region containing 66 protein-coding genes on chromosome 18 (*16*). Among the transcription factors, transport proteins, and enzymes encoded within this region, a gene encoding a predicted BAHD acyltransferase, *AT* (Lalb_Chr18g0051511), was identified as a strong candidate for *pauper* (*17*). The best current marker for *pauper* is located in the coding sequence of *AT* (*18*, *19*). However, this marker does not appear to be a perfect predictor of sweetness, suggesting that *AT* might not be the *pauper* gene (*18*, *20*).

*AT* was first identified together with *LDC* and *CAO* in a differential expression screen between bitter and sweet narrow-leafed lupin (*L. angustifolius* L.) varieties (*21*). LDC and CAO were assigned to the first two steps of the pathway (Fig. 1A), and their activities were later confirmed biochemically (*12*, *13*, *22*). By contrast, AT was putatively assigned to the synthesis of QA esters in the terminal part of the pathway (*21*), but its activity has not been verified. Evidence in support of a more central role for *AT* comes from the fact that the gene is coregulated with *LDC* and *CAO*, while the synthesis of QA esters appears to be decoupled from the core of the pathway (*10*, *23*). Nevertheless, none of the published biosynthetic hypotheses predict any acylation at the core of the QA pathway (*11*), so it is unclear which role *AT* might play there. Intrigued by the conflicting evidence and the prospect of uncovering a novel facet of QA biosynthesis, we decided to investigate whether *AT* really is the *pauper* gene in white lupin.

## RESULTS AND DISCUSSION

Plants bearing the *pauper* allele do not show reduced expression levels of *LDC* and *CAO* compared to bitter plants (*16*), suggesting that the whole pathway is not suppressed. We speculated that the sweetness in *pauper* plants might be due to pathway blockage, which could leave a metabolic footprint in biosynthetic tissues. Thus, we carried out a liquid chromatography–mass spectrometry (LC-MS)–based untargeted metabolite analysis of three white lupin lines: the wild accession Graecus (bitter), the Ethiopian landrace P27174 (bitter), and the modern *pauper* cultivar Amiga (sweet). For the analysis, we chose two types of biosynthetic tissues, leaves and stems. We identified 12 compounds that were present in most samples of Amiga but were absent from the corresponding P27174 and Graecus samples (Fig. 1B and fig. S1). Six of the compounds (Fig. 1B, cyan and orange traces) appeared to be derivatives of the product of CAO (Δ^1^-piperideine, **3**) based on their MS^2^ spectra [fig. S1; MS^2^ fragment of mass/charge ratio (*m/z*) 84]. The other six compounds (Fig. 1B, blue and red traces) appeared to be either tetrahydroanabasine (**5**)—the spontaneous dimerization product of Δ^1^-piperideine (**3**)—or derivatives thereof (fig. S1; MS^2^ fragment of *m*/*z* 167). Overall, the metabolite data suggest that the biosynthesis of QAs is blocked in *pauper* plants at a step immediately downstream of CAO, leading to the accumulation of QA pathway intermediates and their derivatives (“*pauper* metabolites”; Fig. 1B). To further explore this hypothesis, we attempted to reconstruct a similarly blocked QA pathway in planta by transiently coexpressing LDC and CAO in the leaves of *Nicotiana benthamiana*, which does not naturally produce QAs. As predicted, we were able to detect the same *pauper* metabolites from Amiga in the transiently transformed leaves of *N. benthamiana* (Fig. 1B).

**Fig. 2.**
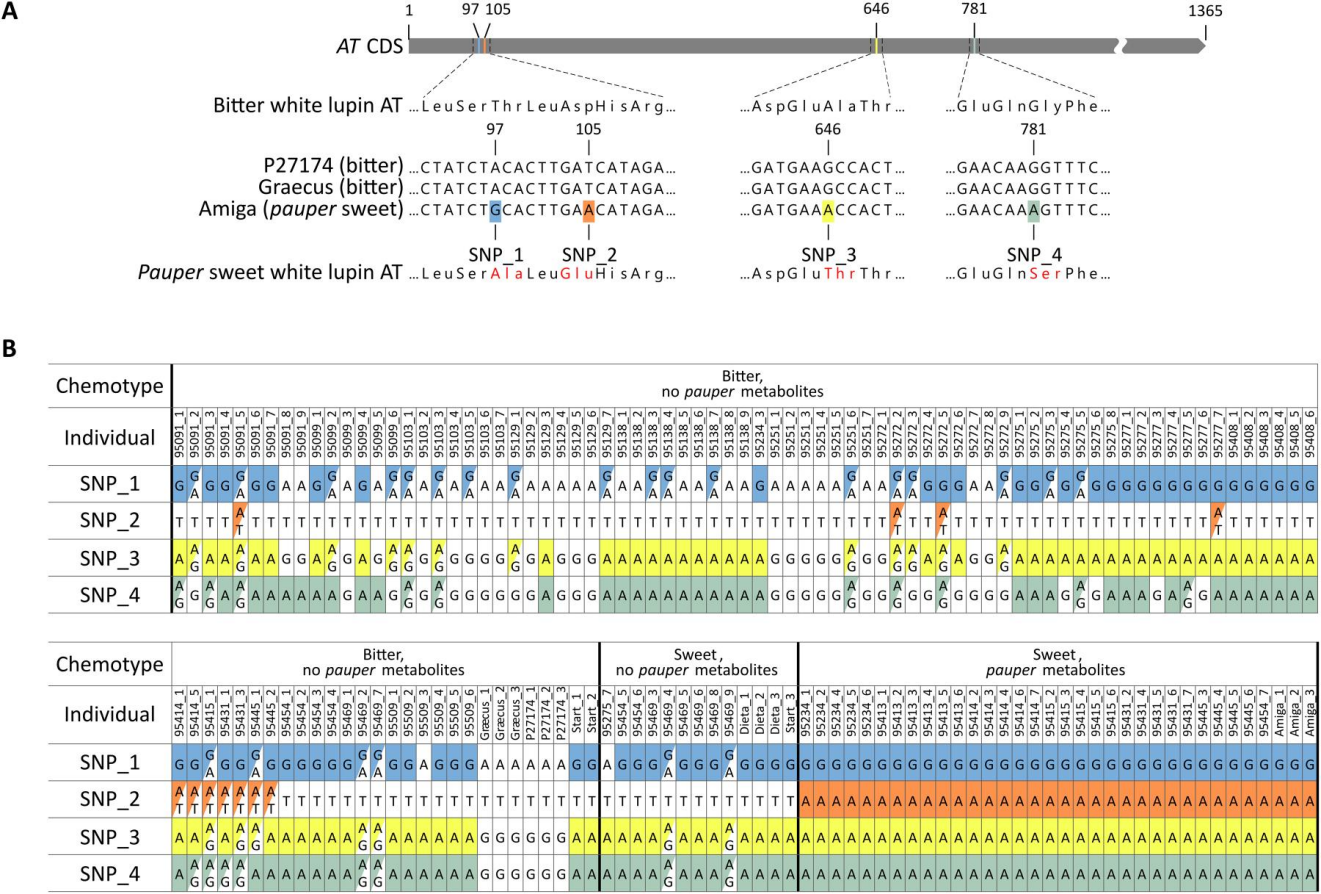
A SNP variant in *AT* is uniquely associated with the *pauper* chemotype. (**A**) Position of four SNPs in the coding sequence of *AT* and their associated variants in Amiga (*pauper* sweet), P27174 (bitter), and Graecus (bitter). The SNP variants found in Amiga (*pauper* sweet) are highlighted with colored backgrounds. (**B**) Leaf chemotype and *AT* SNP variants of a diversity panel derived from 24 white lupin accessions (table S1), as determined by LC-MS and Sanger sequencing. SNP variants identical to those found in Amiga (*pauper* sweet) are highlighted with colored backgrounds [as in (A)], with heterozygous SNPs displaying diagonally split colored backgrounds. The homozygous SNP_2 variant “A” is exclusively associated with the *pauper* sweet chemotype.

We moved to examine whether the affected enzyme could indeed be AT. We compared the *AT* gene sequence from P27174, Graecus, and Amiga and found four single-nucleotide polymorphisms (SNPs) within the coding region (Fig. 2A, DNA Sequences 1 to 3 in the Supplementary Materials). We then used Sanger sequencing and LC-MS to screen a panel composed of 150 individuals derived from a diverse collection of 24 white lupin accessions that had been allowed to cross-pollinate in the field, including bitter, *pauper* sweet, and non-*pauper* sweet accessions (table S1). The results showed a perfect correlation between the Amiga variants at all four SNPs and the *pauper* chemotype (sweetness and accumulation of QA intermediates) (Fig. 2B). The sweet individuals that did not carry the four Amiga SNP variants did not accumulate the *pauper* metabolites (Fig. 2B), suggesting a non-*pauper* sweetness type. We then analyzed a larger panel composed of 227 accessions, which we genotyped by SeqSNP (LGC Biosearch Technologies) and screened for bitterness using a colorimetric test for alkaloids (Dragendorff’s test). The analysis confirmed the complete association between the four Amiga SNP variants and low-alkaloid content in known *pauper* varieties (table S2). In addition, we observed that the Amiga variant at SNP_2 was the only one that was never present in bitter individuals in both panels (Fig. 2B and table S2), suggesting that SNP_2 could be the causal mutation. To address the previous reports of four bitter accessions carrying the Amiga SNP_2 variant (*18*), we obtained three of the four accessions from the same seed collection, genotyped the plants by Sanger sequencing, and analyzed their seeds for alkaloid content by gas chromatography with flame ionization detection (GC-FID). We confirmed that all the plants were bitter; however, none carried the Amiga SNP_2 variant in homozygous state (table S3). Our results also indicate that at least two of these lines were not genetically pure, which could explain the previous conflicting results (table S3).

**Fig. 3.**
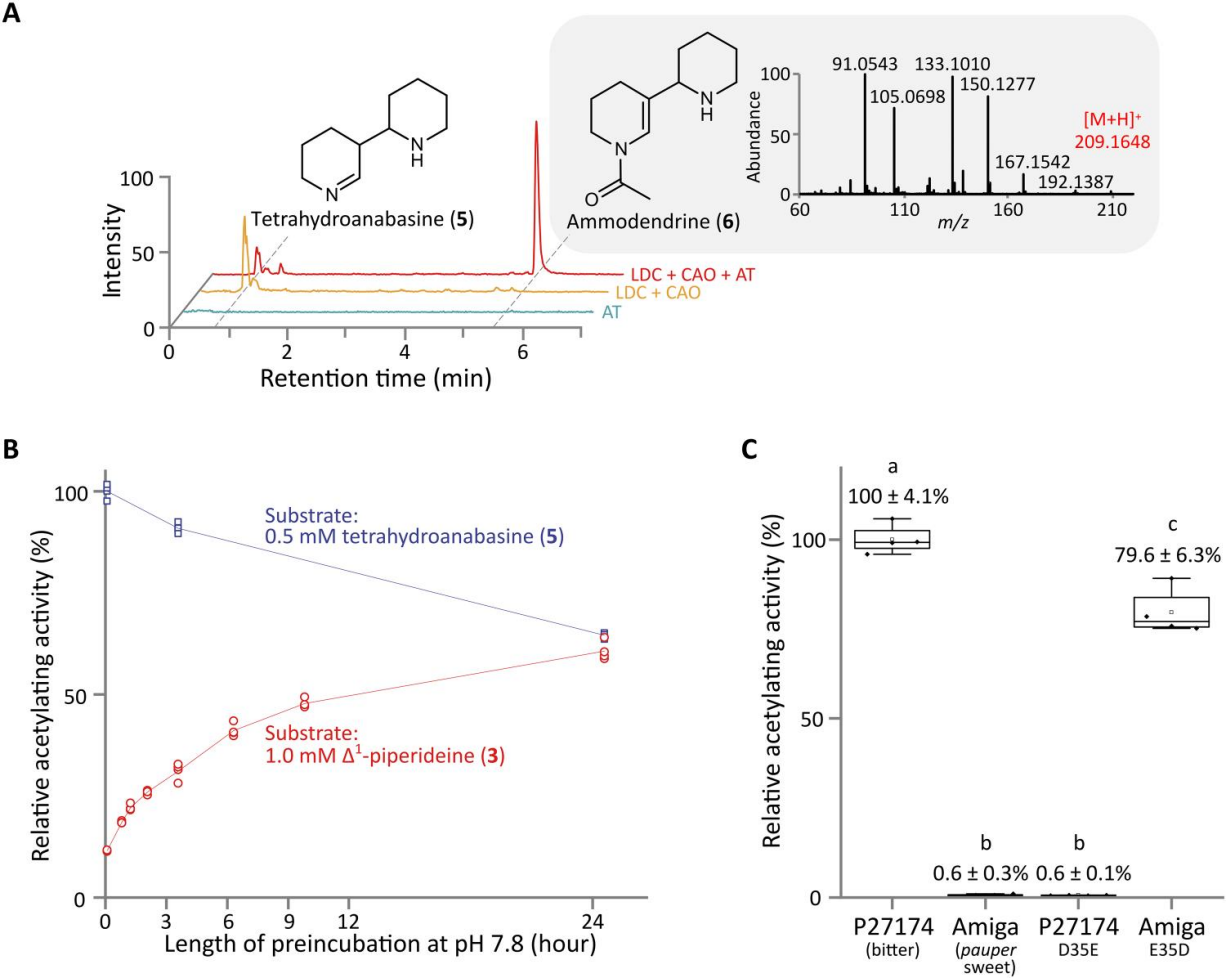
A single SNP in *AT* leads to strongly impaired AT enzyme activity. (**A**) Representative LC-MS chromatograms of extracts from leaves of *N. benthamiana* expressing LDC, CAO, and AT (P27174 bitter version) in different combinations, showing the accumulation of ammodendrine (**6**) and its precursor, tetrahydroanabasine (**5**). MS^2^ spectrum (22.9 eV) of ammodendrine (**6**) is also shown (light gray box). Traces are combined EICs of *m*/*z* 167.15 ± 0.01 and *m*/*z* 209.16 ± 0.01. (**B**) In vitro acetylating activity of AT (P27174 bitter version) against the Δ^1^-piperideine monomer (**3**) and its dimer (tetrahydroanabasine, **5**), showing strong preference for the latter. Monomer and dimer solutions were incubated at pH 7.8 for up to 24 hours before initiating the assay to probe the equilibrium between (**3**) and (**5**) at near-physiological pH. Lines connect the mean relative activities at each time point (*n* = 4). (**C**) In vitro acetylating activity of different versions of AT against tetrahydroanabasine (**5**) showing the effect of the *pauper* variant at SNP_2. In the box plots, the center line represents the median, the box limits represent the upper and lower quartiles, and the whiskers represent maximum and minimum values. Data labels are mean activities relative to P27174 (bitter version) ± 95% confidence interval (*n* = 4). Letters indicate significant differences [one-way analysis of variance (ANOVA) with Tukey’s post hoc test, *P* < 0.05].

We functionally characterized AT in vivo by transiently expressing the version from P27174 in the leaves of *N. benthamiana* together with LDC and CAO. We found that one compound accumulated in large amounts and appeared to be a monoacetylated derivative of tetrahydroanabasine (**5**) (neutral loss of 42 Da giving a fragment of *m*/*z* 167 in MS^2^) (Fig. 3A). We purified the compound and elucidated its structure by nuclear magnetic resonance (NMR) spectroscopy (figs. S3 to S8), which identified it as the bipiperidine alkaloid ammodendrine (**6**) (Fig. 3A and fig. S3). It has long been known that ammodendrine (**6**) derives from cadaverine (*24*) and that it occurs in many species that also produce QAs (*25*). However, to the best of our knowledge, ammodendrine (**6**) has not been proposed as an intermediate in the biosynthesis of QAs. The vast majority of QAs are not *N*-acetylated, which implies that the acetyl group added by AT would have to be removed at a later step in the core QA pathway. Acetylation and subsequent deacetylation are not unprecedented in alkaloid biosynthesis, for example, in the biosynthesis of benzylisoquinoline (*26*) and monoterpene indole alkaloids (*27*, *28*). If acetylation occurs in the early QA pathway, the current biosynthetic pathway proposal (*11*) needs to be amended.

We then developed an absorbance-based assay in microtiter plate format to analyze the AT reaction. We fused the P27174 version of AT to maltose-binding protein (MBP) to improve the solubility of the enzyme (fig. S2A), which enabled expression in *Escherchia coli* and subsequent purification (fig. S2, B to E). Purified MBP-AT was assayed separately against the product of CAO, Δ^1^-piperideine (**3**), and its spontaneously formed dimer, tetrahydroanabasine (**5**). The initial reaction rate was eightfold higher with tetrahydroanabasine (**5**) compared to Δ^1^-piperideine (**3**) (Fig. 3B), suggesting that the dimer is the physiological substrate. Assays with the Amiga version of AT showed a notable impairment in activity down to 0.6% of the P27174 version (Fig. 3C). We then probed the impact of SNP_2 on activity. We observed that introducing the Amiga variant of SNP_2 into P27174 was sufficient to bring the activity of the enzyme down to the same level as the Amiga version itself (Fig. 3C). Conversely, we could rescue most of the activity of the Amiga version by solely exchanging its SNP_2 variant with the one from P27174 (Fig. 3C), which agrees with the results of our diversity panel screens (Fig. 2B and table S2). In Amiga, SNP_2 causes the conservative mutation Asp^35^Glu (Fig. 2A). Asp^35^ is highly conserved in BAHD acyltransferases, and a mutagenesis study in vinorine synthase from *Rauvolfia serpentina* showed a comparable reduction in activity upon exchange with Ala (*29*). Together, our biochemical and genetic results strongly suggest that SNP_2 is indeed the *pauper* mutation.

**Fig. 4.**
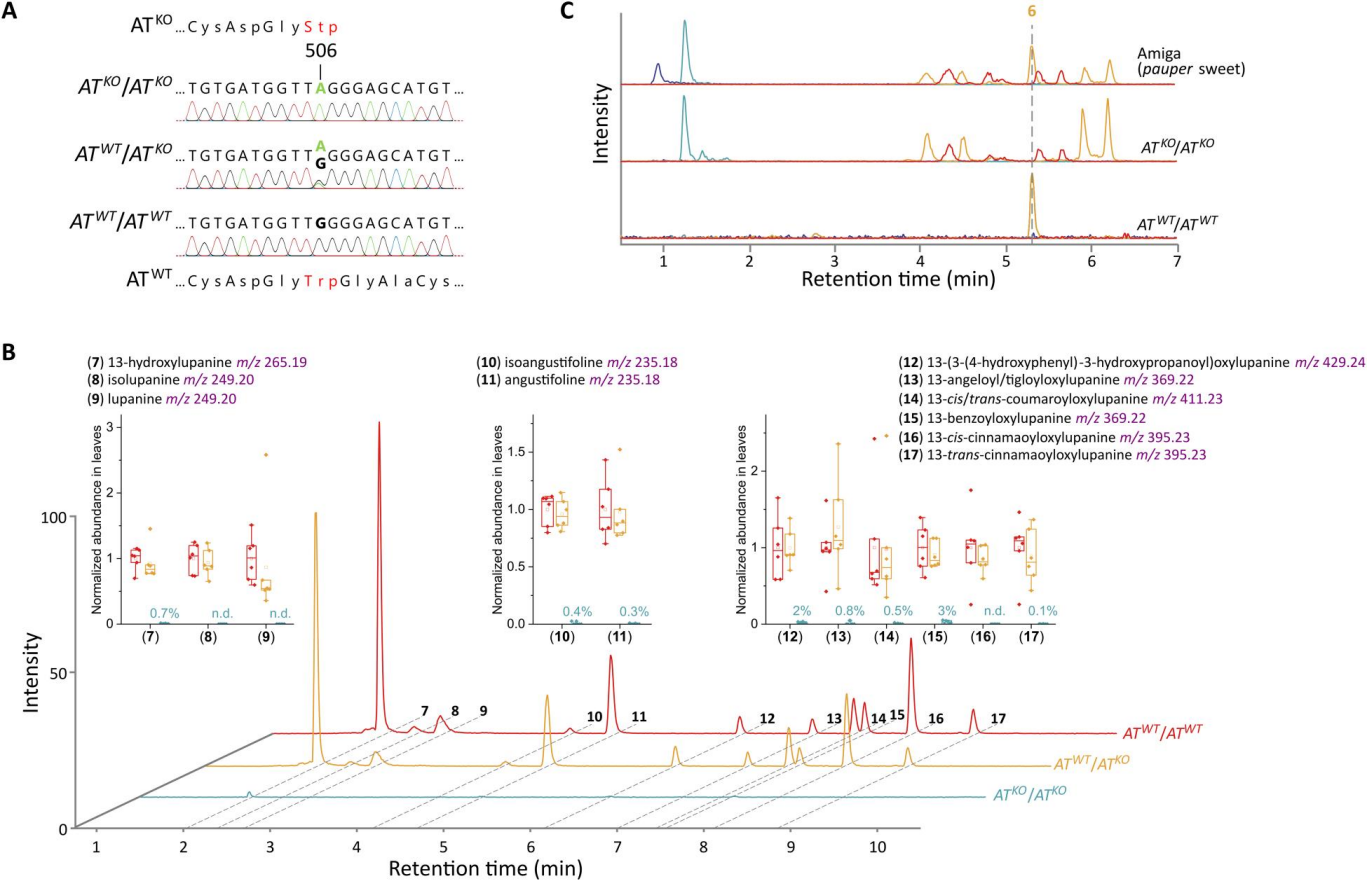
Inactivation of the *AT* gene in narrow-leafed lupin leads to *pauper*-like sweetness. (**A**) Genotyping of mutagenized narrow-leafed lupin plants carrying the wild-type *AT* allele (*AT^WT^*) or an early stop codon allele (*AT^KO^*). (**B**) Distribution and abundance of 11 major QAs (**7** to **17**) in the leaves of narrow-leafed lupin carrying the *AT^WT^* and *AT^KO^* alleles. Traces are combined EICs of *m*/*z* 235.18 ± 0.01, *m*/*z* 249.20 ± 0.01, *m*/*z* 265.19 ± 0.01, *m*/*z* 347.23 ± 0.01, *m*/*z* 369.22 ± 0.01, *m*/*z* 395.23 ± 0.01, *m*/*z* 411.23 ± 0.01, and *m*/*z* 429.24 ± 0.01. In the box plots, the center line represents the median, the box limits represent the upper and lower quartiles, and the whiskers represent maximum and minimum values. Data labels represent the relative abundance (%) in homozygous *AT^KO^* plants relative to homozygous *AT^WT^* plants (*n* = 6). n.d., not detected at the working dilution. (**C**) Representative LC-MS chromatograms showing the accumulation of *pauper* metabolites and the absence of ammodendrine (**6**) in homozygous *AT^KO^* plants. EIC traces correspond to *m*/*z* 167.15 ± 0.01 (blue), *m*/*z* 199.14 ± 0.01 (cyan), *m*/*z* 209.16 ± 0.01 (orange), and *m*/*z* 343.19 ± 0.01 (red).

To confirm the physiological role of *AT*, we built a large mutant library of a bitter cultivar of the related species narrow-leafed lupin. For library construction, we treated around 100,000 seeds (15 kg) with a low dose of mutagen [0.1% ethyl methanesulfonate (EMS)] to minimize the frequency of off-target mutations. We then used FIND-IT technology (*30*) to isolate an early stop codon mutant in the *AT* gene (*AT^KO^*, TGG to TAG at codon 169) (Fig. 4A). The original heterozygous plant did not show a metabolic phenotype (Fig. 4B, upper and middle chromatograms). However, its homozygous mutant progeny displayed foliar sweetness (Fig. 4B, lower chromatogram) and accumulation of *pauper* metabolites (which were undetectable in leaves of the parent bitter cultivar) (Fig. 4C). The *pauper*-like leaf phenotype is consistent with the loss of AT activity at the proposed biosynthetic step (Fig. 1B). Seeds from homozygous mutant plants were also sweet, with a 110-fold reduction in QA levels compared to seeds from wild-type sibling plants (0.028% compared to 3.07% dry seed weight) (table S4). These low QA levels are within the range of *pauper* white lupin cultivars (0.02 to 0.05%) (*14*), confirming that *AT* is an attractive target for the domestication of lupins and other QA-containing legumes. In narrow-leafed lupin, *AT* constitutes a novel sweet gene distinct from the universally used *iucundus* (*31*, *32*) and conferring comparable sweetness (*33*) (table S4). Under growth chamber conditions, the homozygous *AT^KO^* mutant displayed no noticeable phenotype. Originating from chemical mutagenesis, the mutant is exempted from Europe’s strict policy on genetically modified organisms (GMOs) and thus can enter breeding programs without introducing regulatory hurdles.

The discovery of acetylation in early QA biosynthesis has led us to re-examine the current QA pathway hypothesis (*11*). Two theoretical challenges arose during this re-examination. First, our in vitro studies suggested that tetrahydroanabasine (**5**) was the substrate of the acetylation; however, the relevant nitrogen group of **5** is not a likely acetylation center due to weak nucleophilicity. Second, the apparent product of the acetylation, ammodendrine (**6**), has been ruled out as an intermediate in QA biosynthesis due to the absence of a particular hydrogen atom that must be retained during formation of the quinolizidine ring (*11*, *34*). We surmount both hurdles by proposing that the direct substrate of acetylation is not tetrahydroanabasine (**5**) but the carbinolamine **18** (fig. S9), which is likely in equilibrium with **5** under aqueous conditions (*35*–*37*). Accordingly, the direct product of the acetylation would be the carbinolamide **19**, which does retain the mentioned hydrogen atom (fig. S9). In the revised hypothesis, **19** is the true downstream pathway intermediate, while ammodendrine (**6**) is formed spontaneously by dehydration in the absence of the subsequent pathway enzyme. It is worth mentioning that an acetylated QA, lusitanine (**21**), accumulates in several different lupin species (*25*). Its occurrence suggests that the acetyl group is retained at least past the formation of the quinolizidine ring. We summarize the revised pathway hypothesis in fig. S9, which can serve as a starting point for the discovery of the subsequent enzymes in the QA pathway, including a deacetylase responsible for removing the acetyl group.

*AT* is the first sweet gene to be unequivocally identified in lupins. Moreover, the discovery of the causal mutation (SNP_2) provides a mechanistic explanation for the process that enabled the modern domestication of white lupin. The creation of a non-GMO, sweet narrow-leafed lupin line by knocking out the orthologous gene demonstrates the potential to kick-start the domestication of novel legume crops via similar strategies. QAs accumulate not only in the cultivated lupin species but also in around 300 other *Lupinus* species and most other legumes of the wider genistoid clade (*38*). Some of these could become excellent crop candidates upon genetic removal of QAs, for example, the forage legume tagasaste, also known as tree lucerne (*Cytisus proliferus*) (*39*). The use of non-GMO techniques as exemplified here is particularly attractive for legume crop candidates for which transformation protocols are not available and those that are grown in geopolitical areas with restrictive GMO legislation.

## MATERIALS AND METHODS

### LC-MS

LC-MS analyses were carried out on a Thermo Fisher Dionex 3000 RS HPLC/UPLC system interfaced to a Bruker compact QqTOF mass spectrometer through an electrospray ionization (ESI) source. ESI mass spectra (*m*/*z* 50 to 1000) were acquired in positive ionization mode with automatic MS^2^ acquisition using the following parameters: capillary voltage of 4500 V, end plate offset of −500 V, source temperature of 250°C, desolvation gas flow of 8.0 liter/min, and nebulizer pressure of 2.5 bar. N_2_ was used as desolvation, nebulizer, and collision cell gas.

#### LC method 1 (untargeted metabolite analysis in white lupin)

Analytes were separated on a Kinetex XB-C18 column (100 mm by 2.1 mm, 1.7 μm, 100 Å, Phenomenex) kept at 40°C. Mobile phases A and B consisted of, respectively, 0.05% formic acid in water and 0.05% formic acid in acetonitrile. Analytes were eluted using the following gradient at a constant flow rate of 0.3 ml/min: 0 to 1 min, 2% B (constant); 1 to 16 min, 2 to 25% B (linear); 16 to 24 min, 25 to 65% B (linear), 24 to 26 min, 65 to 100% B (linear); 26 to 27 min, 100% B (constant); 27 to 27.5 min, 100 to 2% B (linear); and 27.5 to 33 min, 2% B (constant).

#### LC method 2 (QA quantification in narrow-leafed lupin AT^KO^ plants)

Analytes were separated on a Luna C18(2) column (150 mm by 2 mm, 3 μm, 100 Å, Phenomenex) kept at 40°C. Mobile phases A and B consisted of, respectively, 0.05% formic acid in water and 0.05% formic acid in acetonitrile. Analytes were eluted using the following gradient at a constant flow rate of 0.3 ml/min: 0 to 0.5 min, 2% B (constant); 0.5 to 2.375 min, 2 to 6% B (linear); 2.375 to 7 min, 6 to 25% B (linear), 7 to 13 min, 25 to 100% B (linear); 13 to 14 min, 100% B (constant); 14 to 14.5 min, 100 to 2% B (linear); and 14.5 to 20 min, 2% B (constant).

### Preparative high-performance LC

Preparative high-performance LC (HPLC) was carried out on a Shimadzu preparative HPLC system with ultraviolet detection (190 to 350 nm) and automatic fraction collection. The individual components of the system were: DGU-20A_5_ degasser, SIL-10AP autosampler, LC-20AT pump, CTO-10ASvp column oven, SPD-M20A photodiode array detector, and FRC-10A fraction collector.

Analytes were separated on a Luna C18(2) semi-preparative column (250 mm by 10 mm, 5 μm, 100 Å, Phenomenex) kept at 30°C. Mobile phases A and B consisted of, respectively, 0.05% formic acid in water and 0.05% formic acid in acetonitrile. Analytes were eluted using the following gradient at a constant flow rate of 2 ml/min: 0 to 2 min, 2% B (constant); 2 to 32 min, 2 to 25% B (linear); 32 to 37 min, 25 to 65% B (linear), 37 to 42 min, 65 to 95% B (linear); 42 to 45 min, 95% B (constant); 45 to 50 min, 95 to 5% B (linear); and 50 to 62 min, 2% B (linear). Ammodendrine was collected in the fraction eluting between 21.82 and 22.41 min (λ_max_ = 240 nm).

### Untargeted metabolite analysis of three white lupin accessions (P27174, Graecus, and Amiga)

Seedlings of white lupin accessions P27174 (*n* = 4), Graecus (*n* = 4), and Amiga (*n* = 5) were grown under hydroponic conditions as previously described (*16*). Young leaf material was first collected from the seedlings at the three true leaf stage. The same seedlings were then allowed to grow two more leaves (five true leaf stage). At this point, young leaf and stem material were also harvested. All samples were flash frozen in liquid nitrogen immediately after collection and stored at −50°C until further analysis.

The tissues were pulverized using a mortar and pestle chilled with liquid nitrogen. Five hundred microliters of extractant [60% methanol and 0.06% formic acid in water with caffeine (5 mg/liter) as internal standard] were added to 20 to 100 mg of frozen powder, and the mixtures were shaken vigorously for 2 hours at room temperature. After a brief centrifugation, the extracts were diluted five times with water, clarified through 0.22-μm filters, and transferred to glass vials for LC-MS analysis using LC method 1 (2-μl injections).

Each tissue sample was injected once, and a blank (extractant) and a quality control sample (extract pool) were included every 10th injection to ensure the absence of carryover and to monitor changes in sensitivity during the run. The raw LC-MS chromatograms were mass calibrated (sodium formate clusters), converted to mzXML format, and submitted to XCMS online (v.3.7.1) for peak alignment and metabolite feature detection and integration. The samples were then grouped by accession (P27174, Graecus, and Amiga) and processed together through a multi-job analysis using the default settings for UPLC/Bruker Q-TOF instruments. The parameters used for metabolite feature detection (centWave algorithm) were as follows: Δ *m*/*z* ≤ 10 parts per million (ppm), minimum peak width of 5 s, maximum peak width of 20 s, and signal-to-noise ratio > 5. A total of 6254 metabolite features were detected. Background metabolite features (those detected in the blanks) and those from the calibrant and re-equilibration segments (retention time < 0.5 min and > 27.5 min, respectively) were removed, leaving 3322 lupin metabolite features.

Only eight metabolite features were detected in 9 or more of the 15 tissue samples from Amiga but not in any of the samples from P27174 and Graecus. The eight features corresponded to 17 chromatographic peaks. The median *m*/*z* of the features was as follows: 199.1442 (two peaks), 201.1599 (one peak), 209.1649 (two features, four peaks), 262.1550 (two peaks), 287.0551 (two peaks), 321.6413 (one peak), and 343.1861 (five peaks). The MS^2^ spectrum of all the peaks except those of *m*/*z* 287.0551 (probable flavonoids that were later also found in non-*pauper* accessions) contained a fragment with the mass of Δ^1^-piperideine (**3**) (*m*/*z* 84.08 ± 0.01). The MS^2^ spectrum of the peaks of *m*/*z* 343.1861 and *m*/*z* 321.6413 included also a fragment with the mass of tetrahydroanabasine (**5**) (*m*/*z* 167.16 ± 0.01), dimer of Δ^1^-piperideine (**3**). The most intense of the Amiga-specific peaks were those associated with the features of *m*/*z* 209.1649, *m*/*z* 199.1442, and *m*/*z* 343.1861, in this order. These accumulated in sufficient amounts to be detected in all of the Amiga samples upon visual inspection of the chromatograms. Therefore, only these peaks (peaks b-l in Fig. 1C and fig. S1) were used as metabolic markers for *pauper* in our scoring of white lupin accessions (see below). The compounds likely derive from the oxidation and conjugation of piperideine (**3** or **4**) and tetrahydroanabasine (**5**) in planta, but piperideine itself (**3** or **4**) was not detected in any of the samples. Instead, tetrahydroanabasine (**5**) (median *m*/*z* 167.1542) appeared as a single, broad peak in a fraction of the samples from Amiga (although in lower abundance compared to the others). The scaling factors for the *m*/*z* 167.15, 199.14, 209.16, and 343.19 extracted ion chromatograms in Fig. 1C and fig. S1 are, respectively, 10, 10, 1, and 10 (P27174 and Graecus); 15, 1, 2.5, and 15 (Amiga); and 3, 1, 40, and 15 (*N. benthamiana*).

### Chemo- and genotyping of 150 individuals from 24 white lupin accessions

White lupin accessions were chosen from the Polish *Lupinus* collection to cover a wide range of seed alkaloid levels based on Kroc *et al.* (*14*). The chosen accessions represented different classes (wild forms, landraces, breeding lines, and cultivars) and different countries of origin. Plants were grown in the field at Poznan Plant Breeders Ltd., Wiatrowo, Poland (latitude, 52°45′9″N; longitude, 17°80′36″E; and altitude 86 m above sea level) during the growth seasons of 2016 and 2019. Fifteen seeds per accession were sown in rows during early spring (April), leaving 20 cm between seeds in a row and between rows. Fertilizers P_2_O_5_ and K_2_O were applied at 60 and 90 kg/hectare, respectively, and weed control was carried out mechanically. Leaf samples were collected at flowering stage and immediately frozen for later chemo- and genotyping.

For *AT* genotyping, genomic DNA was extracted from 200 mg of frozen leaf tissue with the aid of the Maxwell RSC Instrument automatic nucleic acid purification platform and the Maxwell RSC Pure Food GMO and Authentication Kit (Promega). Polymerase chain reaction (PCR) was carried out on the extracted DNA using primers LalbAT_F1/R1 and LalbAT_F2/R2 (table S5), and the PCR products were subjected to Sanger sequencing.

For chemotyping, 20 to 70 mg of frozen leaf powder were extracted as described above for the untargeted metabolite analysis of Amiga, P27174, and Graecus and analyzed by LC-MS using LC method 1 (2- and 10-μl injections). Lupanine content was measured using a known standard (Innosil), and the presence of the *pauper* metabolic markers of *m*/*z* 209.16, *m*/*z* 199.14, and *m*/*z* 343.19 (± 0.01) was scored by inspection of the corresponding extracted ion chromatograms (peak signal-to-noise ratio > 5). Samples were labeled as low QA (sweet) if they contained less than 0.02% of lupanine by frozen weight.

### Chemo- and genotyping of 227 white lupin accessions

The white lupin accessions originated from 26 countries and included accessions from six Genebanks and two companies as well as newly collected accessions from Ethiopia. The seeds were chipped and germinated on agar before being vernalized at 4°C for 3 weeks. Seedlings were transferred to soil and grown in a glasshouse under long-day conditions. When plants had five and six true leaves, leaf material was collected for DNA extraction, and samples were tested for QAs by pressing freshly cut petioles onto filter paper soaked in Dragendorff reagent and observing the color change (*40*). DNA was extracted from young leaves using the DNeasy Plant Mini Kit (QIAGEN) and eluted into water. DNA was quantified using NanoDrop and Qubit assays (Thermo Fisher Scientific), and samples were normalized to 50 ng/μl. Purified DNA samples were provided to LGC Genomics (Berlin, Germany) for genotyping by sequencing using SeqSNP, including technical replicate samples for Kiev mutant and P27174. Among the 10,000 SNP targets in the SeqSNP genotyping set were 16 SNPs in the *AT* gene (Chr18g0051511). SeqSNP results are only reported for the four relevant SNPs (see Fig. 2A).

### Chemo- and genotyping of conflicting white lupin accessions

Seed samples of three white lupin accessions were retrieved from the Polish *Lupinus* collection: 95015 (San Felices), 95023 (Oeiras-930/3), and 95064 (Population-8062). For each accession, 10 seeds were sown in the experimental field at the Institute of Plant Genetics, Polish Academy of Sciences, Poznan, Poland (latitude, 52°26′48″N; longitude, 16°54′11″E). Leaf and seed material of all germinated plants underwent genotyping and chemotyping. Genotyping was performed as described above for the chemo- and genotyping of 150 individuals from 24 white lupin accessions. For chemotyping, the total alkaloid content of the mature, dry seeds was determined by GC-FID as previously described (*41*).

### Pathway reconstruction by transient coexpression in *N. benthamiana*

Total RNA was extracted from young leaves of narrow-leafed lupin cv. Oskar and white lupin accession P27174 using the Spectrum Plant Total RNA Kit (Sigma-Aldrich). cDNA was synthesized using the iScript cDNA Synthesis Kit (Bio-Rad). Full-length coding sequences of *LDC* [National Center for Biotechnology Information (NCBI) GenBank AB560664], *CAO* (NCBI GenBank MF152953), and *AT* (DNA Sequence 1 in the Supplementary Materials) were amplified from lupin leaf cDNA (primers in table S5) and cloned into the plant expression vector pEAQ-USER (*42*) by USER cloning (*43*). The constructs were transformed into *Agrobacterium tumefaciens* strain AGL-1. The AGL-1 strains were cultured in 1% yeast extract, 1% peptone medium supplemented with kanamycin (50 μg/ml), rifampicin (25 μg/ml), and carbenicillin (50 μg/ml) at 28°C and 220 rpm to optical density at 600 nm (OD_600_) ≍ 3. The presence of the desired pEAQ-USER construct was confirmed by culture PCR. Following a brief centrifugation, bacterial pellets were resuspended in water to OD_600_ = 1. After a 1- to 3-hour incubation at room temperature, the bacterial suspensions were infiltrated into the abaxial side of the young leaves of 4- to 5-week-old *N. benthamiana* plants using a 3-ml syringe without a needle. To coexpress multiple genes in the same leaf, suspensions of the corresponding AGL-1 strains were mixed in equal volumes before the infiltration. Only the youngest, fully expanded leaf of each plant was infiltrated, and three to five plants were used for each combination of genes.

Agroinfiltrated leaves were harvested 9 days after infiltration. Two 1-cm leaf discs were punched out of the infiltrated (slightly discolored) portion of the leaves and flash-frozen in liquid nitrogen. The discs were pulverized using steel balls and a TissueLyser II bead beater (QIAGEN). The pulverized leaf discs were extracted with 250 μl of extractant [60% methanol and 0.06% formic acid in water with caffeine (5 mg/liter) as internal standard] by vigorously shaking for 2 hours at room temperature. The solids were separated by centrifugation, and the supernatants were diluted 5× with water, passed through 0.22-μm filters, and transferred to glass vials for LC-MS analysis using LC method 1.

### Purification and structural elucidation of the AT product from *N. benthamiana*

LDC, CAO, and AT were coexpressed in young leaves from 4-week-old *N. benthamiana* plants by agroinfiltration as described above. Whole infiltrated leaves were harvested 7 days after the agroinfiltration, quickly frozen in liquid nitrogen, and pulverized. A total of 5 ml of extractant (60% methanol and 0.06% formic acid in water) were added to 800 mg of frozen leaf powder. The mixture was shaken for 2 hours at room temperature. The light green mixture was centrifuged, and the supernatant was concentrated under vacuum at room temperature to a viscous yellow oil. The oily residue was diluted with 2 ml of 20% methanol in water and passed through a 0.22-μm filter.

Ammodendrine was isolated from the extracts as its formate salt by preparative HPLC (200-μl injections). The ammodendrine fractions from up to 40 consecutive injections were pooled and dried at 35°C under vacuum to a white, powdery residue. The residue was dissolved in D_2_O with 0.05% sodium 2,2-dimethyl-2-silapentane-5-sulfonate (DSS) as internal reference (calibrated to δ_H_ 0.0 ppm and δ_C_ 0.0 ppm) and analyzed by NMR spectroscopy. NMR spectra were acquired at 300 K on a Bruker Avance III spectrometer (^1^H operating frequency of 600.13 MHz) equipped with a Bruker SampleJet sample changer and a cryogenically cooled gradient inverse triple resonance 1.7-mm TCI probe-head (Bruker Biospin, Karlsruhe, Germany) using standard one-dimensional (1D) and 2D experiments [^13^C NMR data were assigned using heteronuclear single quantum coherence (HSQC) and heteronuclear multiple bond correlation (HMBC) experiments]. Data were processed using TopSpin version 4.1.4 (Bruker). Ammodendrine (**6**) appeared as an equimolar mixture of *cis*:*trans* rotamers about the amide bond in D_2_O.

#### cis-Ammodendrine

^1^H NMR (600 MHz, D_2_O, 300 K, DSS): δ_H_ 6.90 (1H, s, H6), 3.67 (1H, m, H2′ax), 3.63 (1H, m, H2A), 3.52 (1H, ddd, J = 13.0, 7.7, 3.7 Hz, H2B), 3.41 (1H, d, *J* = 11.4 Hz, H6′eq), 3.06 (1H, t, *J* ≍ 12 Hz, 6′ax), 2.20 (3H, s, H8), 2.18 (1H, m, H4A), 2.10 (1H, m, H4B), 1.94 (1H, m, H4’eq), 1.92 (1H, m, H3′eq), 1.89 (1H, m, H5′eq), 1.86 (2H, m, H3), 1.78 (1H, m, H3′ax), 1.65 (1H, m, H5′ax), and 1.58 (1H, m, H4′ax). ^13^C NMR (150 MHz, D2O, 300 K, DSS): δ_C_ 174.9 (C7), 128.8 (C6), 120.3 (C5), 63.7 (C2′), 47.9 (C6′), 43.0 (C2), 30.1 (C3′), 24.7 (C4′), 24.3 (C5′), 23.8 (C4), 23.3 (C8), and 23.1 (C3).

#### trans-Ammodendrine

^1^H NMR (600 MHz, D_2_O, 300 K, DSS): δ_H_ 7.23 (1H, s, H6), 3.69 (1H, m, H2′ax), 3.66 (1H, m, H2A), 3.59 (1H, m, H2B), 3.41 (1H, d, J = 11.4 Hz, H6′eq), 3.06 (1H, t, J ≍ 12 Hz, H6′ax), 2.19 (3H, s, H8), 2.18 (1H, m, H4A), 2.10 (1H, m, H4B), 1.94 (1H, m, H4′eq), 1.92 (1H, m, H3′eq), 1.92 (2H, m, H3), 1.89 (1H, m, H5′eq), 1.78 (1H, m, H3′ax), 1.65 (1H, m, H5′ax), and 1.58 (1H, m, H4′ax). ^13^C NMR (150 MHz, D_2_O, 300 K, DSS): δ_C_ 174.9 (C7), 125.9 (C6), 121.7 (C5), 63.7 (C2′), 47.9 (C6′), 46.9 (C2), 30.1 (C3′), 24.7 (C4′), 24.3 (C5′), 23.9 (C4), 23.7 (C8), and 23.6 (C3).

High-resolution mass of [M+H]^+^ calculated for C_12_H_21_N_2_O: 209.1648; found 209.1649 (err. −0.3 ppm).

### Chemical synthesis of α-tripiperideine and tetrahydroanabasine

The two different trimers of Δ^1^-piperideine (**3**), α-tripiperideine and isotripiperideine, were synthesized as previously described (*44*). Tetrahydroanabasine was prepared as the hydrobromide salt of its carbinolamine form using isotripiperdiene as a starting point, as previously described (*37*).

### Heterologous protein expression, purification, and assays

Full-length sequences of the *AT* versionsfrom accessions Amiga and P27174 were codon-optimized for expression in *E. coli* (see DNA Sequences 4 and 5 in the Supplementary Materials). The codon-optimized sequences were synthesized and cloned between the Nde I and Xho I restriction sites of the expression vector pET28a(+) by Twist Bioscience. However, N-terminally His-tagged AT proved to be insoluble when expressed in *E. coli* in our initial tests. To increase solubility, the enzyme was fused to the common solubility tag, *E. coli* MBP. A custom T7-lac-based expression cassette was designed to encode a cleavable (by HRV 3C protease), N-terminally His-tagged MBP tag upstream of a USER cloning site (fig. S8A and DNA Sequence 6 in the Supplementary Materials). The cassette was synthesized and cloned between the Bgl II and Xho I restriction sites of expression vector pET24a(+) by Twist Bioscience. The resulting new plasmid (pET24-HMP-USER) was amplified in *E. coli* strain TOP10 and digested with Pac I and Nt.BbvC I. The codon-optimized *AT* versions from Amiga and P27174 were then cloned by USER cloning (*43*) in frame with the N-terminal tags using the primers specified in table S5. The point mutations leading to the Amiga^E35D^ and P27174^D35E^ *AT* versions were introduced in the respective codon-optimized sequences via USER fusion (*45*) using an additional primer pair carrying the desired mutation for each version (table S5).

The four *AT* constructs in pET24-HMP-USER were transformed into *E. coli* strain ArcticExpress (DE3) RIL. The ArcticExpress strains were precultured overnight at 37°C and 220 rpm in selective LB [gentamicin (20 μg/ml), streptomycin (75 μg/ml), tetracycline (10 μg/ml), and kanamycin (50 μg/ml)] from fresh, single colonies. A 2-ml aliquot of preculture was added to 100 ml of terrific broth [yeast extract (24 g/liter), tryptone (20 g/liter), glycerol (4 ml/liter), 17 mM KH_2_PO_4_, and 72 mM K_2_HPO_4_ (pH 7.2)] without antibiotics and incubated at 30°C and 220 rpm to OD_600_ ≍ 0.6 to 0.8. The cultures were briefly cooled down in an ice-water bath, and the AT expression was induced with 0.250 mM isopropyl-β-D-thiogalactopyranoside (IPTG). The cultures were incubated at 10°C and 220 rpm for 60 to 90 hours after which bacterial pellets were resuspended in 6 ml of ice-cold lysis buffer [300 mM NaCl, 10% glycerol, 50 mM Na_2_HPO_4_, and lysozyme (100 μg/ml) (pH 8.0)]. The cells were lysed by sonication on ice-water, and the lysates were cleared by centrifugation at 4°C and 20,000 rcf for 20 min. For affinity purification, the cleared lysates were incubated with 1.5 ml of 50% Ni-NTA agarose resin (QIAGEN) for 1 hour at 4°C on a tube rotator. The matrix was washed with 12 ml of ice-cold wash buffer [300 mM NaCl, 10% glycerol, 50 mM Na_2_HPO_4_, and 25 mM Imidazole (pH 8.0)]. Bound proteins were eluted with 4.5 ml of ice-cold elution buffer [300 mM NaCl, 10% glycerol, 50 mM Na_2_HPO_4_, and 500 mM imidazole (pH 8.0)]. Imidazole was removed via Amicon Ultra MWCO 30 kDa diafiltration centrifugal columns (Merck), which also served to concentrate the protein 90× using exchange buffer [300 mM NaCl, 10% glycerol, and 50 mM Na_2_HPO_4_ (pH 8.0)]. The final concentration of tagged AT was determined by in-gel quantification using Criterion TGX strain free gels (Bio-Rad) and a bovine serum albumin (BSA) standard (Bio-Rad). The protein preparations could be stored at 4°C for up to 15 days with ~50% loss in activity. See also Extended Methods in Supplementary Materials.

To measure the rate of the acetylation reactions, we developed an absorption-based assay in microtiter plate format. In the assay, coenzyme A produced by AT reacted quantitatively and in real time with the thiol scavenger and chromogen 5,5'-dithiobis(2-nitrobenzoic acid) (DTNB) to form a yellow compound (λ_max_ = 412 nm). Individual reactions were carried out in a final volume of 200 μl and contained 100 mM Na_2_HPO_4_ (pH 8.0), 500 μM DTNB (Sigma-Aldrich), 1 mM acetyl-coenzyme A trilithium salt (Roche), 1 mM Δ^1^-piperideine (prepared in house from α-tripiperideine; see Extended Methods in the Supplementary Materials), and 5 to 50 μg of tagged AT. In alternative assays, the Δ^1^-piperideine was replaced by 0.5 mM tetrahydroanabasine dihydrobromide. Care was taken not to mix AT with acetyl-coenzyme A and DTNB before the start of the assay, as doing so appeared to inhibit the enzyme. Assays were started by adding 195 μl of a mixture of substrates and DTNB to 5-μl aliquots of the protein, and absorbance at 412 nm was monitored for 10 min at room temperature using a microplate reader with 3-s shaking between each reading. See Extended Methods for additional details.

### Narrow-leafed lupin mutant library construction

The narrow-leafed lupin mutant library was constructed essentially as described in Knudsen *et al.* (*30*). Briefly, 15 kg of narrow-leafed lupin cultivar Oskar was subjected to mutagenesis using 0.1% EMS for 16 hours at 23°C. The EMS was removed by three successive washes with water before the seeds were fan dried on filter paper in a fume hood. The dried M1 seeds were subsequently shipped to New Zealand for field propagation and harvest. Plants were harvested in 5-m^2^ pools using a combine harvester. Genomic DNA was extracted from subsamples of each pool of M2 seeds, and the remaining pool was stored until seed extraction.

### Isolation and characterization of a narrow-leafed lupin *AT* knockout

The narrow-leafed lupin mutant library was screened essentially as described in Knudsen *et al.* (*30*). Briefly, a TaqMan assay was designed to identify a specific base change at position 506 in the coding region of *AT*, which corresponds to the change in amino acid 169 from tryptophan to a premature stop (see DNA Sequence 7 in the Supplementary Materials). For the TaqMan assay, we designed a target-specific forward primer (AT_TaqMan_FW), a target-specific reverse primer (AT_TaqMan_RV), a wild-type–specific probe containing both a HEX fluorophore and a BHQ1 quencher (ATWT_TaqMan_HEX), and a mutant-specific probe containing both the FAM fluorophore and a BHQ1 quencher (ATKO_TaqMan_FAM). The TaqMan assay was used to screen the pools of the narrow-leafed lupin mutant DNA library utilizing the FIND-IT workflow (*30*). Subsequently, one individual heterozygous mutant seed was extracted from a positive pool of the library by subjecting 768 individual seeds to a nondestructive DNA extraction procedure as described previously for barley (*30*).

The M2 heterozygous mutant plant that grew from the seed was allowed to self-pollinate. The resulting M3 seeds were sown in 16-cm-wide, 20-cm-deep pots filled with commercial peat-based potting soil and grown in a growth cabinet with a light/dark photoperiod of 16/8 hours at day/night temperatures of 21°/18°C and at 60% relative humidity. Young leaves from the M3 plants were dried for 12 hours at 50°C and pulverized using steel balls and a bead beater. Genomic DNA was extracted from ~5 mg of dry leaf powder using the E.Z.N.A. Plant DNA DS Kit (Omega Bio-tek), and the plants were subsequently genotyped by sequencing a 315-bp-long PCR fragment spanning the early stop codon using primers ATKO_GnTp_FW and ATKO_GnTp_RV. QAs were extracted from ~10 mg of dry leaf powder with 1 ml of extractant (60% methanol and 0.06% formic acid in water with 15-ppm caffeine as internal standard) by mixing vigorously for 2.5 hours at room temperature. The extracts were clarified by centrifugation, diluted 15× with water, and filtered through a 0.22-μm filter. The samples were analyzed by LC-MS using LC method 2 (1-μl injections). The identity of the alkaloids was inferred from their *m*/*z* and MS^2^ fragmentation spectrum (*22*). For relative quantification, QA peak areas were normalized by the area of the caffeine peak and by the weight of the sample (*n* = 6 for each genotype). The total alkaloid content of the mature, dry seeds of the M3 plants was determined by GC-FID as previously described (*41*).

## Supplementary Materials

### This PDF file includes:

Extended Methods DNA Sequences 1 to 7 Figs. S1 to S9 Legends for tables S1 to S5 References
