# Supplementary material for: The causal mutation leading to sweetness in modern white lupin cultivars
Source: Sci Adv. 2023 Aug 4;9(31):eadg8866. doi: 10.1126/sciadv.adg8866 (PMC10403207; doi:10.1126/sciadv.adg8866)
Supplement: Supplementary file 1 — Extended Methods DNA Sequences 1 to 7 Figs. S1 to S9 Legends for tables S1 to S5 References [file sciadv.adg8866_sm.pdf]

Science Advances
AAAS

Supplementary Materials for

The causal mutation leading to sweetness in modern white lupin cultivars

Davide Mancinotti *et al.*

Corresponding author: Fernando Geu-Flores, feg@plen.ku.dk

Sci. Adv. **9**, eadg8866 (2023)
DOI: 10.1126/sciadv.adg8866

The PDF file includes:

Extended Methods
DNA Sequences 1 to 7
Figs. S1 to S9
Legends for tables S1 to S5
References

Other Supplementary Material for this manuscript includes the following:

Tables S1 to S5

Extended methods

Heterologous expression and purification of AT

For regular maintenance, the ArcticExpress (DE3) RIL expression strains carrying the pET24-HMP-AT constructs were cultured on low-salt LB medium supplemented with 20 µg/mL gentamicin, 75 µg/mL streptomycin, 10 µg/mL tetracycline, and 50 µg/mL kanamycin. For expression, single colonies were inoculated in 5 mL selective LB and grown overnight at 37 °C and 220 rpm in 50 ml centrifuge tubes. 2 mL of the overnight cultures were added to 100 mL terrific broth without antibiotics (24 g/L yeast extract, 20 g/L tryptone, 4 mL/L glycerol, 17 mM KH_2PO_4, 72 mM K_2HPO_4, pH 7.2) in 1-L conical flasks. The cultures were incubated at 30 °C and 220 rpm until OD_{600} ≈ 0.6-0.8 (≈2-3 hours) and placed on ice for 15 min. 25 µL of 1 M isopropyl β-D-1-thiogalactopyranoside (IPTG) were added to the cultures to induce the expression of the recombinant proteins, and the cultures were incubated at 10 °C and 220 rpm for 60-90 hours. The bacteria were harvested by centrifugation for at 3000 rcf for 10 min at 4 °C and the bacterial pellets were resuspended in 6 mL ice-cold lysis buffer (300 mM NaCl, 10% glycerol, 100 mM Na_2HPO_4, 100 µg/mL lysozyme, pH 8.0) by vortexing. The mixtures were sonicated at 300-400 W on water-ice for 6x 10 s with 30-s pauses. The lysates were transferred to 2 mL microcentrifuge tubes and centrifuged for at 20000 rcf for 20 min at 4 °C. 1.5 mL of 50% Ni-NTA agarose resin (Qiagen) were equilibrated with lysis buffer in 15 mL centrifuge tubes. The clarified lysates were added to the equilibrated resin and incubated at 4 °C on a tube rotator with gentle rotation (15 rpm) for 1 hour. The samples were loaded onto 3 mL fritted spin columns (G-Biosciences) and the lysate was allowed to flow through by gravity. The resin was then washed with 12 mL of ice-cold wash buffer (300 mM NaCl, 10% glycerol, 50 mM Na_2HPO_4, 25 mM imidazole, pH 8.0). Bound proteins were eluted with 3 x 1.5 mL portions of ice-cold elution buffer (300 mM NaCl, 10% glycerol, 50 mM Na_2HPO_4, 500 mM imidazole, pH 8.0). The pooled elutes were loaded onto 4 mL Amicon® Ultra MWCO 30 kDa diafiltration centrifugal columns (Merck), concentrated to 50 µL by centrifugation (3000 rcf for 30 min at 4 °C), diluted with 450 µL ice-cold buffer without imidazole (300 mM NaCl, 10% glycerol, 50 mM Na_2HPO_4, pH 8.0), and concentrated again to 50 µL. The process was repeated 2 more times. The concentration of tagged AT in the protein preparations (25x dilutions) was determined by in-gel quantification using Criterion™ TGX strain free gels (Bio-Rad) and a BSA standard (Bio-Rad).

Time-course dimerization of piperideine

To probe the substrate specificity of AT and the equilibration between piperideine and tetrahydroanabasine, solutions of piperideine and tetrahydroanabasine in an aqueous buffer at pH 7.8 were incubated at room temperature for different lengths of time and then assayed using AT. The 1 mM Δ^1-piperideine substrate

mixtures were prepared by mixing in 1.5 mL microcentrifuge tubes 746 µL of assay buffer (100 mM Na_2HPO_4 buffer, pH 8) and 2.2 µL of 133 mM α-tripiperideine in 2 M HCl (final pH 7.8). The 0.5 mM tetrahydroanabasine substrate mixtures were prepared by mixing in 1.5 mL microcentrifuge tubes 702 µL of assay buffer, 2.2 µL of 2 M HCl, and 44 µL 10 mM tetrahydroanabasine hydrobromide in assay buffer. All samples were incubated at room temperature in the dark for the required amount of time. Eight Δ^1-piperideine mixtures and three tetrahydroanabasine mixtures were prepared in the course of the 24 hours preceding the start of the assay. 30 min before the start of the assay, 88 µL of 10 mM acetyl coenzyme A trilithium salt (Roche) in assay buffer and 22 µL of 20 mM DTNB (Sigma-Aldrich) in assay buffer were added to every substrate mixture. 5 µL aliquots of 1 µg/µL tagged $LaAT^{P27174}$ (in 300 mM NaCl, 10% glycerol, 50 mM Na_2HPO_4, pH 8.0) were distributed in the wells of a 96-well clear, flat-bottom polystyrene plate (Thermo Scientific - Nunc). The enzymatic reactions were started by adding 195 µL of the piperideine or tetrahydroanabasine substrate mixtures to the wells (*n* = 4 for each mixture). As a blank, we mixed boiled (15 min at 95 °C) tagged $LaAT^{P27174}$ (in 300 mM NaCl, 10% glycerol, 50 mM Na_2HPO_4, pH 8.0) with freshly prepared 0.5 mM tetrahydroanabasine substrate (*n* = 4). The absorbance at 412 nm was read on a plate reader with 15 s intervals and 3 s shaking between reads. Initial reaction velocities were estimated by linear regression on the first five datapoints.

Test of the AT variants in vitro

To compare the activity of the four AT variants, the purified enzymes were assayed against the preferred substrate tetrahydroanabasine. A tetrahydroanabasine substrate mixture was prepared by mixing in a 5-mL centrifuge tube 4160 µL of assay buffer (100 mM Na_2HPO_4 buffer, pH 8), 520 µL of 10 mM acetyl coenzyme A trilithium salt (Roche) in assay buffer, 130 µL of 20 mM 5,5'-dithio-bis-(2-nitrobenzoic acid) (DTNB) (Sigma-Aldrich) in assay buffer, and 260 µL of 10 mM tetrahydroanabasine hydrobromide in assay buffer. 5 µL aliquots of 2 µg/µL tagged AT^{P27174} and AT^{Amiga_E35D} and 5 µl aliquots of 10 µg/µL tagged AT^{Amiga} and AT^{P27174_D35E} (in 300 mM NaCl, 10% glycerol, 50 mM Na_2HPO_4, pH 8.0) were distributed in the wells of a 96-well clear, flat-bottom polystyrene plate (Thermo Scientific - Nunc). Boiled (15 min at 95 °C) tagged AT variants (in 300 mM NaCl, 10% glycerol, 50 mM Na_2HPO_4, pH 8.0) were used as blanks (*n* = 1 for each AT variant). The enzymatic reactions were started by adding 195 µL of the substrate mixture to the wells (*n* = 4 for each AT variant). The absorbance was read at 412 nm on a plate reader with 9 s intervals and 3 s shaking between reads. Initial reaction velocities were estimated by linear regression on the first five datapoints.

DNA Sequence 1. Native coding sequence of AT variant from P27174 (bitter white lupin landrace).

ATGGCATATCAAATGGCATCACTGAAAATTGAGATGAAAGAAGTAGTGCATGTTAAACCTTCTAAACCAACTCCTTCC
ATTGTTCTTCCTCTATCTACACTTGATCATAGACCCTATCCTGATAGCATTTGGCCTATTGTTCATGTTTACCAATCACCC
TCAAATGGCCAACTAGATCCTGCTTTTGTGCTCAAACAAGCCCTCTCAAAGGCTTTGGTTTATTATTACCCTCTTGCAGG
TAAGCTAGTAAAACAACCCAACGGAAAAGTTGCTATCAATTGCAACAATGATGGAGTTCCATTCTTGGAAGCAATTGC
AAATTGTGAGCTTTCATCTCTCAATTATCTAGATGGTCATGACATTCGAATTGCAAAACAATTGGTTTTTGATTTTCATC
CTCAACAAGATGAAAATGAATACCCACATCCAGTTTCATTCAAGTTGACCAAATTCCAATGTGGAGGTTTCACAATTGG
AATGAGCACATCACATATTGTATGTGATGGTTGGGGAGCATGTAAGTTCTTCCATGCCATTGTTGAACTTGCAAGTGG
AAAAAGTGAACCCTTTTTGAAACCTGTTTGGGAGAGAGAAAGATTAATAGGATCAATCACTACACAACCAATGCCAAA
TCCAATGGATGAAGCCACTGCTGCAGTTTCACCATTTCTTCCAGCCACTGATGTTATGTATGAGTTGTTTAAGGTGGAC
AAGGAGAGTATAAGAAGACTCAAGATGAGTTTAATGAAGGAAATTAGTTGCAATGAATCAATGGAACAAGGTTTCAC
AACATTTGAATCTCTTGCTGCATATGTCTGGAGATCAAGAGCTAGGGCCTTAAACCTAAATAATGAAGGGAAAACTTT
GCTTGTTTTCTCAGTGCAGGTGAGACAACACATGAGTCCTCCTTTGTCTGATGGGTACTATGGAACTGCTATCACAGAA
GGACAAGTTGTGCTAACCATGAAGGAGCTCAATGAGAAACCACTCTCAGATATAGTGAAGCTTGTCAAAGAGAGTAA
AAATGTTGCATTCACTGGTGATTTTATCAAAAAAACAATTGATACATTGGAGTCTAATCCAGAGAATTTTAATGTTGAA
GAAGGTCCTGGTGCAACCTTGGCTTTATCAGATTGGAAGCATTTAGGTTTCATGCCAAATGTGGATTTTGGATGGAAG
GAACCAATAAATATGGTACCTGCTCCATGCAACATGTTTGAGTATGAGGGTTTGTGCATTTTCTTGTCTCCTAGTAACC
ATGATCCATCAATGGAAGGAGGAGTTAGGGTTTTCATATCACTCCCTAGTGTTGCCATGCCTAAGTTTAAAGAGGAGA
TGGAAGCTCTCAAGGTTATTACACCTTAG

DNA Sequence 2. Native coding sequence of AT variant from Graecus (bitter white lupin wild accession).

ATGGCATATCAAATGGCATCACTGAAAATTGAGATGAAAGAAGTAGTGCATGTTAAACCTTCTAAACCAACTCCTTCC
TTGTTCTTCCTCTATCTACACTTGATCATAGACCCTATCCTGATAGCATTTGGCCTATTGTTCATGTTTACCAATCACCCT
CAAATGGCCAACTAGATCCTGCTTTTGTGCTCAAACAAGCCCTCTCAAAGGCTTTGGTTTATTATTACCCTCTTGCAGGT
AAGCTAGTAAAACAACCCAACGGAAAAGTTGCTATCAATTGCAACAATGATGGAGTTCCATTCTTGGAAGCAATTGCA
AATTGTGAGCTTTCATCTCTCAATTATCTAGATGATCATGACATTCGAATTGCAAAACAATTGGTTTTTGATTTTCATCCT
CAACAAGATGAAAATGAATACCCACATCCAGTTTCATTCAAGTTGACCAAATTCCAATGTGGAGGTTTCACAATTGGA
ATGAGCACATCACATATTGTATGTGATGGTTGGGGAGCATGTAAGTTCTTCCATGCCATTGTTGAACTTGCAAGTGGA
AAAAGTGAACCCTTTTTGAAACCTGTTTGGGAGAGAGAAAGATTAATAGGATCAATCACTACACAACCAATGCCAAAT
CCAATGGATGAAGCCACTGCTGCAGTTTCACCATTTCTTCCAGCCACTGATGTTATGTATGAGTTGTTTAAGGTGGACA
AGGAGAGTATAAGAAGACTCAAGATGAGTTTAATGAAGGAAATTAGTTGCAATGAATCAATGGAACAAGGTTTCACA
ACATTTGAATCTCTTGCTGCATATGTCTGGAGATCAAGAGCTAGGGCCTTAAACCTAAATAATGAAGGGAAAACTTTG
CTTGTTTTCTCAGTGCAGGTGAGACAACACATGAGTCCTCCTTTGTCTGATGGGTACTATGGAACTGCTATCACAGAAG
GACAAGTTGTGCTAACCATGAAGGAGCTCAATGAGAAACCACTCTCAGATATAGTGAAGCTTGTCAAAGAGAGTAAA
AATGTTGCATTCACTGGTGATTTTATCAAAAAAACAATTGATACATTGGAGTCTAATCCAGAGAATTTTAATGTTGAAG
AAGGTCCTGGTGCAACCTTGGCTTTATCAGATTGGAAGCATTTAGGTTTCATGCCAAATGTGGATTTTGGATGGAAGG
AACCAATAAATATGGTACCTGCTCCATGCAACATGTTTGAGTATGAGGGTTTGTGCATTTTCTTGTCTCCTAGTAACCAT
GATCCATCAATGGAAGGAGGAGTTAGGGTTTTCATATCACTCCCTAGTGTTGCCATGCCTAAGTTTAAAGAGGAGATG
GAAGCTCTCAAGGTTATTACACCTTAG

DNA Sequence 3. Native coding sequence of AT variant from Amiga (*pauper* sweet white lupin cultivar).

ATGGCATATCAAATGGCATCACTGAAAATTGAGATGAAAGAAGTAGTGCATGTTAAACCTTCTAAACCAACTCCTTCC
ATTGTTCTTCCTCTATCTGCACTTGAACATAGACCCTATCCTGATAGCATTTGGCCTATTGTTCATGTTTACCAATCACCC
TCAAATGGCCAACTAGATCCTGCTTTTGTGCTCAAACAAGCCCTCTCAAAGGCATTGGTTTATTATTACCCTCTTGCAGG
TAAGCTAGTAAAACAACCCAACGGAAAAGTTGCTATCAATTGCAACAATGATGGAGTTCCATTCTTGGAAGCAATTGC
AAATTGTGAGCTTTCATCTCTCAATTATCTAGATGATCATGACATTCGAATTGCAAAACAATTGGTTTTTGATTTTCATC
CTCAACAAGATGAAAATGAATACCCACATCCAGTTTCATTCAAGTTGACCAAATTCCAATGTGGAGGTTTCACAATTGG
AATGAGCACATCACATATTGTATGTGATGGTTGGGGAGCATGTAAGTTCTTCCATGCCATTGTTGAACTTGCAAGTGG
AAAAAGTGAACCCTTTTTGAAACCTGTTTGGGAGAGAGAAAGATTAATAGGATCAATCACTACACAACCAATGCCAAA
TCCAATGGATGAAACCACTGCTGCAGTTTCACCATTTCTTCCAGCCACTGATGTTATGTATGAGTTGTTTAAGGTGGAC
AAGGAGAGTATAAGAAGACTCAAGATGAGTTTAATGAAGGAAATTAGTTGCAATGAATCAATGGAACAAAGTTTCAC
AACATTTGAATCTCTTGCTGCATATGTCTGGAGATCAAGAGCTAGGGCCTTAAACCTAAATAATGAAGGGAAAACTTT
GCTTGTTTTCTCAGTGCAGGTGAGACAACACATGAGTCCTCCTTTGTCTGATGGGTACTATGGAACTGCTATCACAGAA
GGACAAGTTGTGCTAACCATGAAGGAGCTCAATGAGAAACCACTCTCAGATATAGTGAAGCTTGTCAAAGAGAGTAA
AAATGTTGCATTCACTGGTGATTTTATCAAAAAAACAATTGATACATTGGAGTCTAATCCAGAGAATTTTAATGTTGAA
GAAGGTCCTGGTGCAACCTTGGCTTTATCAGATTGGAAGCATTTAGGTTTCATGCCAAATGTGGATTTTGGATGGAAG
GAACCAATAAATATGGTACCTGCTCCATGCAACATGTTTGAGTATGAGGGTTTGTGCATTTTCTTGTCTCCTAGTAACC
ATGATCCATCAATGGAAGGAGGAGTTAGGGTTTTCATATCACTCCCTAGTGTTGCCATGCCTAAGTTTAAAGAGGAGA
TGGAAGCTCTCAAGGTTATTACACCTTAG

DNA Sequence 4. ***E. coli*** **codon-optimized sequence of AT variant from Amiga (*pauper* sweet white lupin cultivar).**

ATGGCTTACCAGATGGCCAGCCTGAAGATCGAAATGAAGGAAGTTGTACACGTGAAGCCGAGCAAGCCGACCCCGTC
TATCGTACTCCCATTAAGCGCGCTGGAGCACCGTCCTTACCCCGACTCCATCTGGCCGATCGTGCACGTATATCAGAGT
CCGTCGAACGGGCAGTTAGACCCGGCGTTCGTCCTGAAGCAGGCGTTATCTAAAGCCCTTGTATACTACTATCCACTC
GCTGGCAAACTTGTTAAGCAGCCTAATGGTAAGGTGGCGATTAACTGTAATAACGACGGCGTCCCGTTTCTTGAGGCC
ATCGCCAACTGCGAACTGAGCAGCTTGAACTACCTGGACGACCACGATATCCGCATCGCGAAGCAGCTCGTGTTCGAC
TTCCACCCGCAGCAGGACGAGAACGAGTATCCGCACCCGGTGTCGTTTAAACTGACGAAGTTTCAGTGCGGTGGCTTT
ACGATCGGCATGTCTACCAGCCACATCGTGTGCGACGGCTGGGGTGCCTGCAAATTCTTTCACGCGATCGTGGAGTTG
GCGTCGGGCAAGTCTGAGCCATTCCTGAAGCCAGTCTGGGAACGCGAGCGTCTGATTGGCTCCATTACGACCCAGCC
GATGCCGAACCCTATGGACGAGACTACCGCGGCTGTATCTCCCTTTCTCCCGGCTACCGACGTCATGTACGAATTATTC
AAAGTCGATAAAGAATCTATTCGTCGCCTTAAAATGAGCCTGATGAAAGAGATCAGCTGTAACGAGAGTATGGAGCA
GTCCTTTACCACCTTCGAGTCCCTCGCAGCCTACGTGTGGCGCTCTCGTGCCCGCGCGCTGAATCTGAACAACGAGGG
CAAGACATTACTGGTATTTTCGGTCCAAGTTCGCCAGCACATGTCGCCGCCCCTGAGTGACGGTTATTACGGGACGGC
CATTACCGAGGGGCAGGTCGTCTTGACTATGAAAGAACTGAACGAAAAGCCGTTGTCCGACATTGTAAAATTAGTGA
AGGAAAGCAAGAACGTGGCGTTTACGGGCGACTTCATTAAGAAGACCATCGACACGTTAGAATCGAACCCGGAAAAC
TTCAACGTCGAAGAGGGGCCGGGCGCTACGCTGGCCCTCTCTGACTGGAAACACCTGGGCTTTATGCCTAACGTCGAC
TTCGGCTGGAAAGAGCCCATTAACATGGTGCCAGCCCCGTGTAATATGTTCGAATACGAAGGCCTGTGTATCTTTCTG
TCACCGTCTAATCACGACCCCTCGATGGAAGGCGGTGTACGCGTGTTTATTTCCCTGCCGTCAGTGGCTATGCCGAAAT
TCAAGGAAGAAATGGAAGCCCTGAAAGTGATCACCCCCTAA

DNA Sequence 5. ***E. coli*** **codon-optimized sequence of AT variant from P27174 (bitter white lupin landrace).**

ATGGCTTACCAGATGGCTAGCTTAAAGATCGAAATGAAGGAAGTGGTCCACGTAAAGCCGTCCAAGCCGACACCGAG
CATCGTGCTCCCGTTAAGTACTTTGGACCACCGCCCGTACCCGGACAGTATCTGGCCGATCGTGCACGTCTATCAGTCT
CCAAGCAACGGGCAGCTGGACCCGGCGTTCGTTCTGAAGCAGGCGCTTAGCAAAGCCCTGGTGTACTACTATCCACT
GGCGGGCAAACTGGTTAAGCAGCCGAATGGTAAGGTCGCAATTAACTGTAATAACGACGGCGTGCCTTTCTTAGAGG
CCATCGCTAACTGCGAATTGAGCAGTCTGAACTACCTTGACGGGCACGATATCCGCATCGCCAAGCAGCTGGTCTTCG
ACTTCCACCCGCAGCAGGACGAGAACGAGTATCCGCACCCGGTCTCCTTTAAACTGACGAAGTTTCAGTGCGGTGGAT
TTACGATCGGCATGTCCACGAGCCACATCGTGTGCGACGGATGGGGCGCTTGCAAATTCTTTCACGCGATCGTGGAGT
TGGCGTCGGGTAAGTCAGAGCCATTCTTAAAGCCAGTGTGGGAACGCGAGCGCCTTATTGGGAGCATTACCACCCAG
CCGATGCCGAACCCTATGGACGAGGCTACCGCGGCGGTATCCCCTTTCCTGCCTGCAACCGACGTAATGTACGAACTG
TTCAAAGTTGATAAAGAAAGCATTCGCCGCCTGAAAATGAGCTTGATGAAAGAGATCTCTTGTAACGAGAGCATGGA
GCAGGGGTTTACTACCTTCGAGTCCTTAGCCGCGTACGTGTGGCGTAGCCGTGCCCGCGCACTGAATCTGAACAACGA
GGGAAAGACCCTGCTGGTATTTTCTGTACAAGTTCGCCAGCACATGAGCCCGCCGCTGTCGGACGGCTATTACGGCAC
CGCGATTACCGAGGGCCAGGTAGTACTGACGATGAAAGAATTGAACGAAAAGCCGCTGTCGGACATTGTCAAACTCG
TGAAGGAAAGCAAGAACGTCGCGTTTACGGGCGACTTCATTAAGAAGACTATCGACACTCTGGAATCGAACCCGGAA
AACTTCAACGTGGAAGAGGGCCCGGGCGCGACGCTGGCACTTTCGGACTGGAAACACCTGGGCTTTATGCCGAACGT
TGACTTCGGTTGGAAAGAGCCGATTAACATGGTGCCGGCGCCGTGTAATATGTTCGAATACGAAGGACTTTGTATCTT
TCTGTCCCCGTCGAATCACGACCCGTCGATGGAAGGCGGGGTGCGTGTATTTATCAGCCTGCCAAGCGTGGCGATGC
CCAAATTCAAGGAAGAAATGGAAGCACTGAAAGTCATCACGCCGTGA

DNA Sequence 6. Expression cassette of pET24-HMP-USER. The highlighted segments are: the BglII and XhoI consensus sequences in grey, the ribosome binding site in cyan, the start codon in red, and the USER cloning cassette (PacI-Nt.BbvCI) in yellow.

AGATCTCGATCCCGCGAAATTAATACGACTCACTATAGGGGAATTGTGAGCGGATAACAATTCCCCTCTAGAAATAAT
TTTGTTTAACTTTAAGAAGGAGATATACCATGGGCAGCAGCCACCATCATCACCACCATAGCAGCGGCAAAATCGAAG
AAGGTAAACTGGTAATCTGGATTAACGGCGATAAAGGCTATAACGGTCTCGCTGAAGTCGGTAAGAAATTCGAGAAA
GATACCGGAATTAAAGTCACCGTTGAGCATCCGGATAAACTGGAAGAGAAATTCCCACAGGTTGCGGCAACTGGCGA
TGGCCCTGACATTATCTTCTGGGCACACGACCGCTTTGGTGGCTACGCTCAATCTGGCCTGTTGGCTGAAATCACCCCG
GACAAAGCGTTCCAGGACAAGCTGTATCCGTTTACCTGGGATGCCGTACGTTACAACGGCAAGCTGATTGCTTACCCG
ATCGCTGTTGAAGCGTTATCGCTGATTTATAACAAAGACCTGCTGCCGAACCCGCCAAAAACCTGGGAAGAGATCCCG
GCGCTGGATAAAGAACTGAAAGCGAAAGGTAAGAGCGCGCTGATGTTCAACCTGCAAGAACCGTACTTCACCTGGCC
GCTGATTGCTGCTGACGGGGGTTATGCGTTCAAGTATGAAAACGGCAAGTACGACATTAAAGACGTGGGCGTGGATA
ACGCTGGCGCGAAAGCGGGTCTGACCTTCCTGGTTGACCTGATTAAAAACAAACACATGAATGCAGACACCGATTACT
CCATCGCAGAAGCTGCCTTTAATAAAGGCGAAACAGCGATGACCATCAACGGCCCGTGGGCATGGTCCAACATCGAC
ACCAGCAAAGTGAATTATGGTGTAACGGTACTGCCGACCTTCAAGGGTCAACCATCCAAACCGTTCGTTGGCGTGCTG
AGCGCAGGTATTAACGCCGCCAGTCCGAACAAAGAGCTGGCAAAAGAGTTCCTCGAAAACTATCTGCTGACTGATGA
AGGTCTGGAAGCGGTTAATAAAGACAAACCGCTGGGTGCCGTAGCGCTGAAGTCTTACGAGGAAGAGTTGGTGAAA
GATCCGCGTATTGCCGCCACTATGGAAAACGCCCAGAAAGGTGAAATCATGCCGAACATCCCGCAGATGTCCGCTTTC
TGGTATGCCGTGCGTACTGCGGTGATCAACGCCGCCAGCGGTCGTCAGACTGTCGATGAAGCCCTGAAAGACGCGCA
GACTGGAGGCGGTGGATCTGGCGGAGGTGGTAGCCTGGAAGTTCTGTTCCAGGGGCCCGCTGAGGCCTTAATTAATC
CTCAGCAACTCGAG

DNA Sequence 7. Coding sequence of AT variant from Oskar (bitter narrow-leafed lupin cultivar). The wild-type (AT^{WT}) and mutant (AT^{KO}) variants at position 506 are highlighted in cyan and red, respectively.

ATGGCATATCAAATGGCATCACTGAAACTTGAGATGAATGAAGTAGTGCATGTCAAACCTTCTACACCAACACCTTCCA
TTGTTCTTCCTCTATCTACCCTTGACCATAGACCCTATCCTGATAGCATTTGGCCTATAGTTCATGTTTACCGGTCAGCCT
CAAATGGGAAGCTAGATCCTGCTTTTGTGCTCAAACAAGCCCTTTCAAAGGCTTTGGTTTATTATTACCCTCTTGCAGG
TAAGCTAGTAAAACAACCCGACGGAAAAGTTGCTATCAATTGCAACAATGATGGAGTTCCATTCCTGGAAGCAATTGC
AAATTGTAATCTTTCCTCTCTTAATTATCTAGATGATCATGACATCCTAATTGCAAAACAATTGGTTTTCGATTTACATGT
TCAAGATGAAAATGAATACCCACATCCAGTTTCGTTCAAGTTGACCAAATTCCAATGTGGAGGTTTCACAATTGGAATG
AGCACATCACATATTGTGTGTGATGGTT(G/A)GGGAGCATGTCAGTTCTTCCGAGCCATTGTTGAACTGGCAAGTGGT
AAAAGTGAGCCCTTTGTGAAACCTGTTTGGGAGAGAGAAAGATTAATAGGATCAATCACTACACAACCAATGCCAAAT
CCAATGGATGAGGCTACTGCTGCAGTTTCACCATTTCTTCCAGCCACTGATGTTATGTATGAGTTGTTTAAGGTTGACA
AGGAAAGCATAAGAAGACTCAAGATGAGTTTAATGAAGGAAATTAGTGGCAATGAAACAATGGAACAAGGCTTCAC
AAGTTTTGAATCTCTTGCTGCATATGTGTGGAGATCAAGAGCAAGGGCCTTAAACCTAAATAATGAAGGGAAAACTTT
GCTTGTTTTCTCAGTGCAGGTGAGACAACACATGAGTCCTCCTTTATCTGATGGGTACTATGGAACTGCTATCACAGAA
GGGCAAGTTGTGCTAACCATGAAGGAGCTCAATGAGAAACCACTCTCAGATATAGTGAAGCTTGTCAAAGAGAGTAA
AAATATTGCTTTCACTGGTGATTTTATCAAAAACACAATTGATACATTGGAGTCTAATCCAGAGAATTTTAATGTTGAA
GAAGGTCCTGGTGCAACATTGGCTTTATCAGATTGGAAGCATTTGGGTTTCATGCCAAATGTGGATTTTGGATGGAAG
GAACCAATAAATATGGTACCTGCTCCATGCAACATGTTTGAGTATGAGGGTTTGTGCATTTTCTTGTCTCCTAGTAAGT
ATGACCCATCAATGGAAGGAGGAGTTAGGGTTTTCATATCACTCCCTAGTGTTGCCATGCCTAAGTTTAGAGAGGAGA
TGGAAGCTCTGAAGGTTACTACACCCTAG

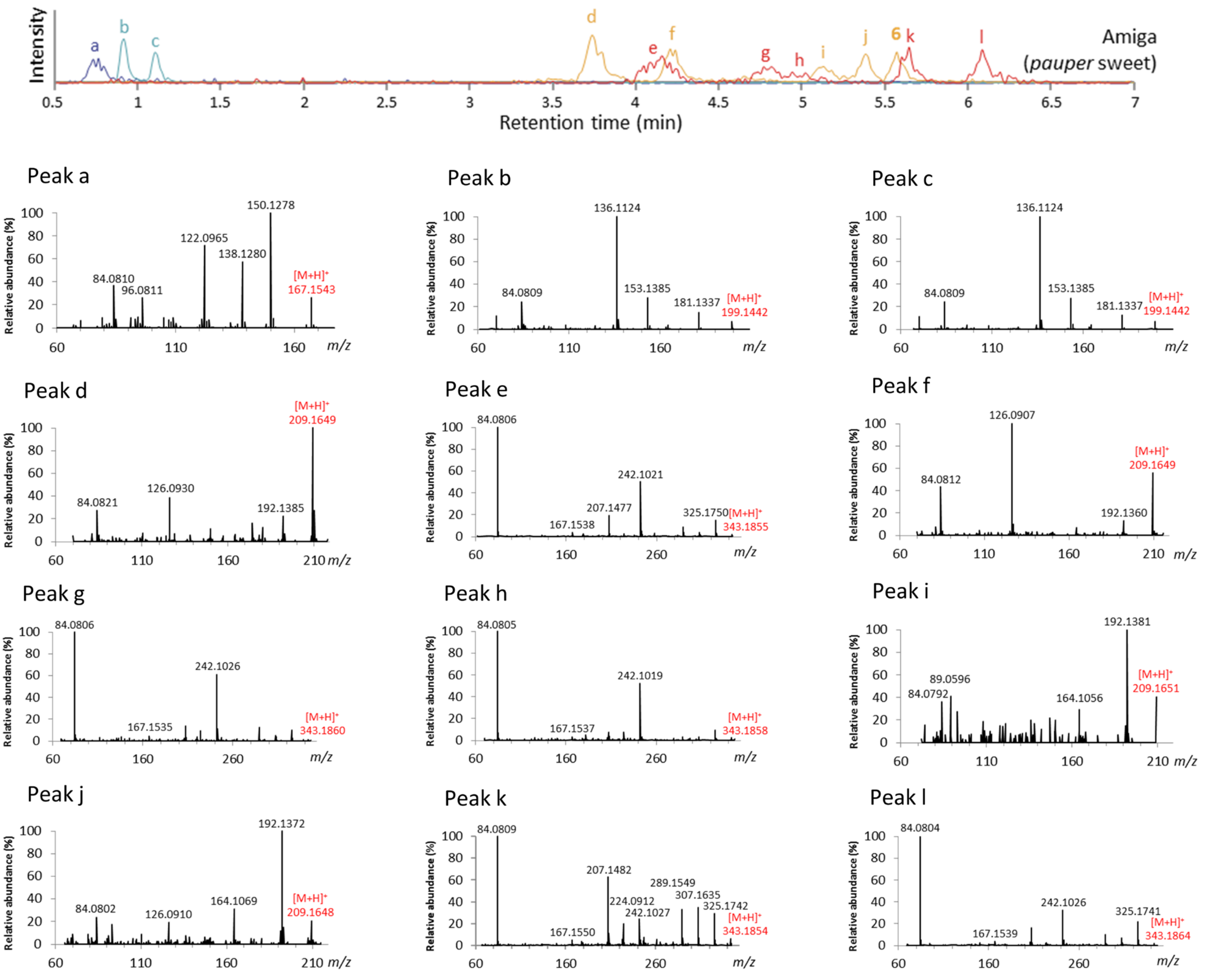

Fig. S1. Collision-induced dissociation (CID) MS^2 spectra of the metabolic markers for *pauper*. A representative liquid chromatography-mass spectrometry (LC-MS) chromatogram of an Amiga leaf extract is shown at the top. Traces are extracted ion chromatograms (EICs) of the most representative *pauper*-specific metabolite features (mean *m/z* ± 0.01). Signal intensities were adjusted to aid visualization (see Materials and Methods for scaling factors). MS^2 spectra were recorded at collision energies of 21.0 eV (peak a), 22.5 eV (peak b and c), 22.9 eV (peak d, f, i, and j), and 28.1 eV (peak e, g, h, k, and l). The numerical labels in the MS^2 spectra indicate the *m/z* values of parent molecular ions (in red), fragments of relative abundance above 20%, and fragments that indicate a piperideine (*m/z* 84.08) or tetrahydroanabasine (*m/z* 167.15) moiety. Peak 6 is ammodendrine (**6**) (MS^2 spectrum in Fig. 3A), which is not a metabolic marker for *pauper*.

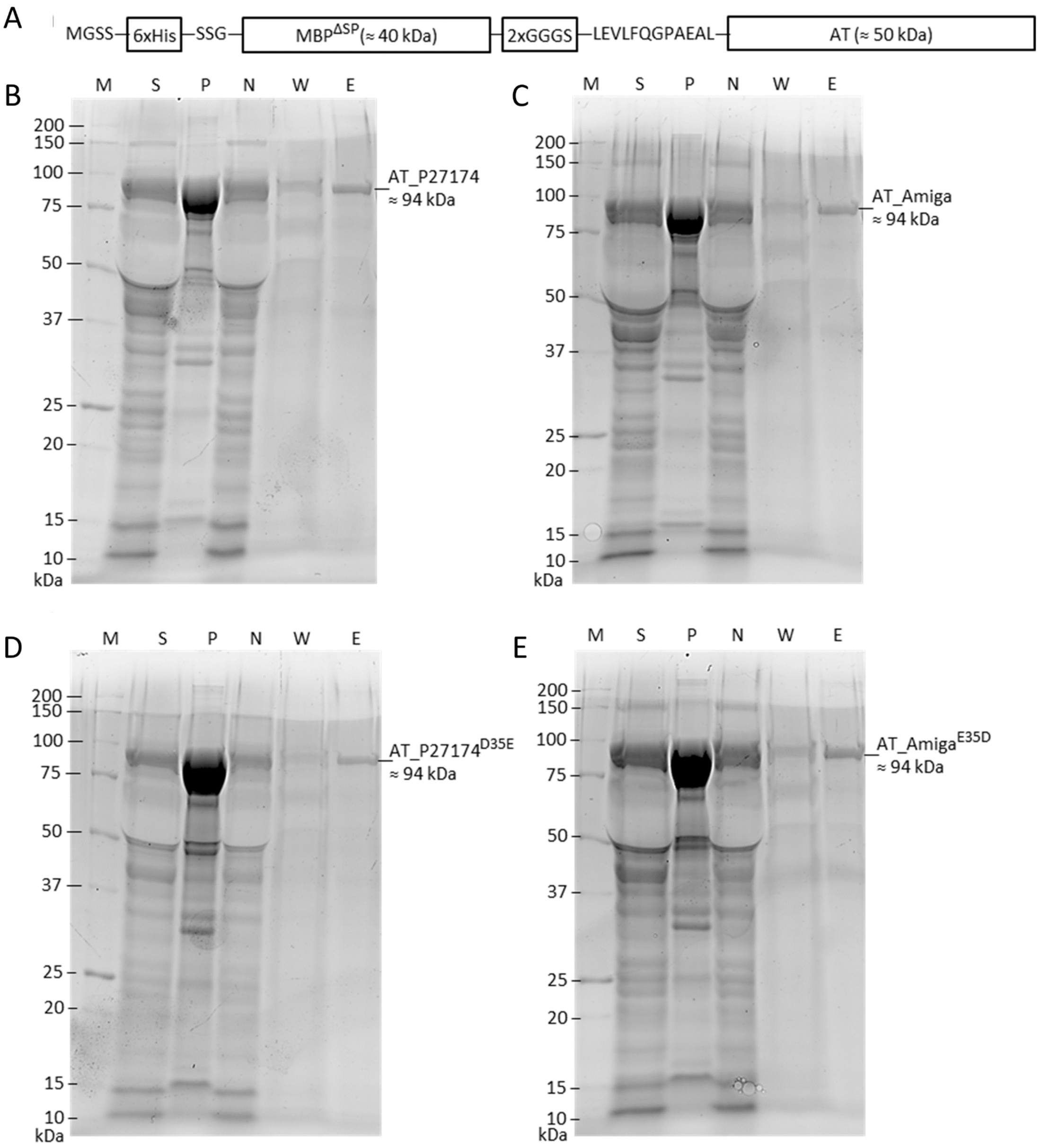

Fig. S2. Expression of AT variants in *E. coli*. (**A**) structure of the His-MBP-tagged AT proteins expressed from the custom vector pET24-HMP-USER. On the N-terminus, the His-tag is separated from the MBP-tag by a very short flexible spacer (SSG). A longer, flexible spacer (2xGGGS) then separates $MBP^{\Delta SP}$ from a HRV 3C protease cleavage site (LEVLFQ'GP) and the proper AT protein. The remaining amino acids (AEAL) immediately upstream of AT are due to the introduction of the USER cloning cassette. Approximate molecular weights are shown for the $MBP^{\Delta SP}$ and the AT components. $MBP^{\Delta SP}$: maltose-binding protein from *E. coli* without periplasm targeting signal. (**B** to **E**) AT was expressed in the *E. coli* strain ArcticExpress (DE3) RIL at 10 °C for 60-90 hours (here 90 hours) and purified by affinity chromatography on a Ni-NTA matrix. The yields of purified, tagged AT protein from this preparation were 1.5 to 4 mg for a 100 mL culture. M: molecular weight marker; S: soluble fraction of the bacterial lysate; P: insoluble fraction of the bacterial lysate; N: proteins not bound to the Ni-NTA resin; W: wash (25 mM imidazole); E: eluate (500 mM imidazole).

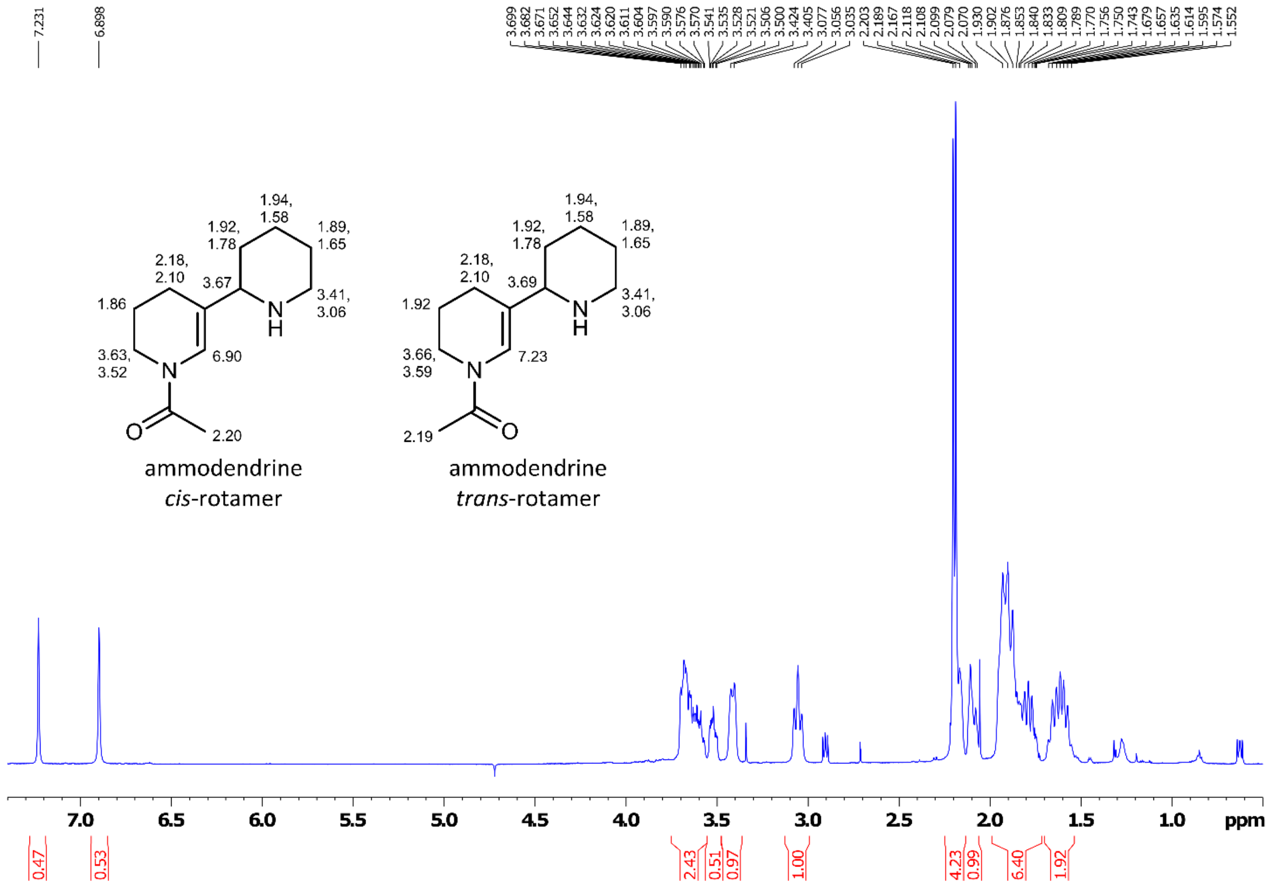

Fig. S3. 1D ^{1}H NOESY spectrum of ammodendrine (6) formate (600 MHz, D_2O, 300 K, DSS). ^{1}H resonances are relative to the trimethylsilyl group of DSS (not shown) and the experiment was acquired with presaturation at 4.7 ppm to remove the HDO resonance. The formate proton resonance downfield (singlet, 8.44 ppm) is not shown. The smaller, not integrated signals are due to impurities and DSS.

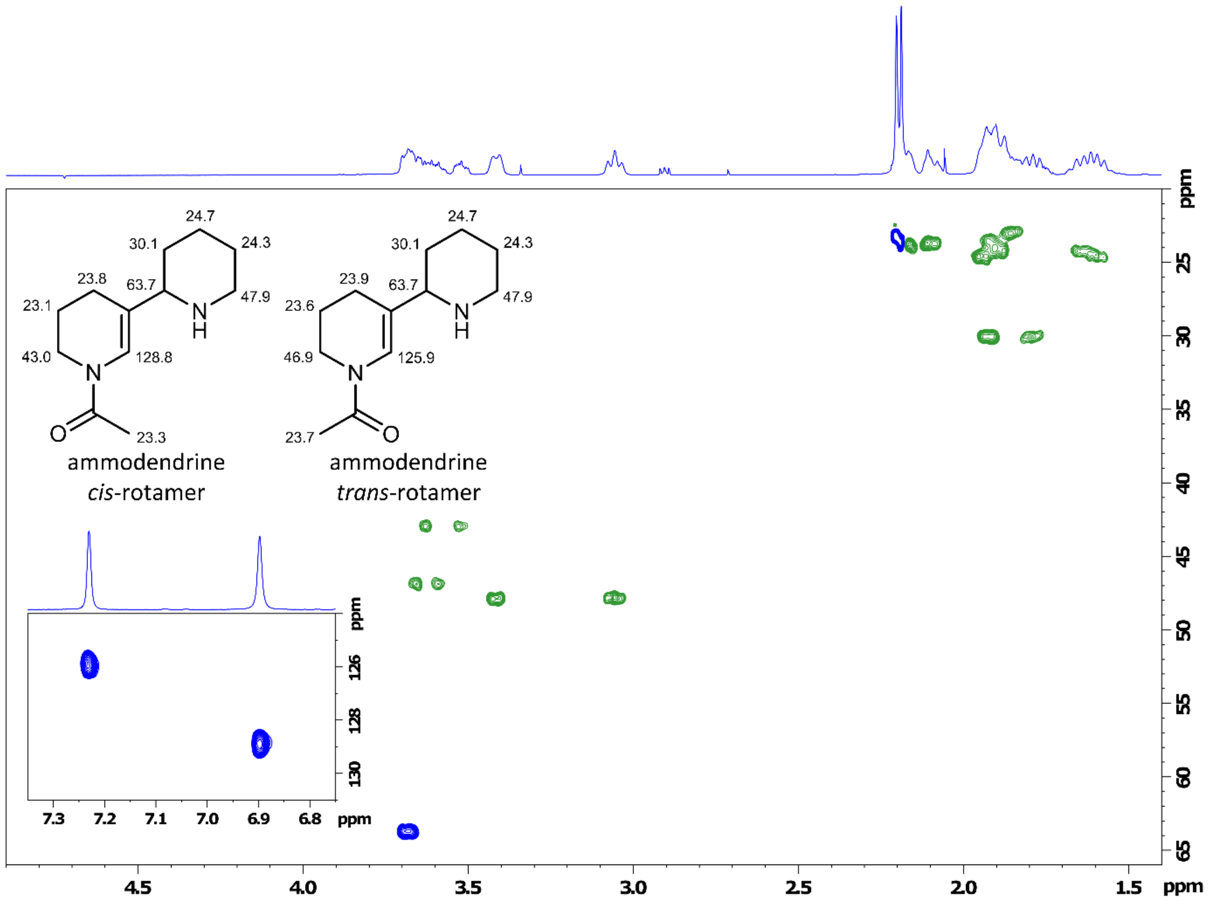

Fig. S4. ^{1}H-^{13}C HSQC spectrum of ammodendrine (6) formate (600 MHz, D_2O, 300 K, DSS). ^{13}C resonances are relative to the trimethylsilyl group of DSS (not shown). The formate correlation downfield (8.44 ppm and 173.8 ppm) is not shown.

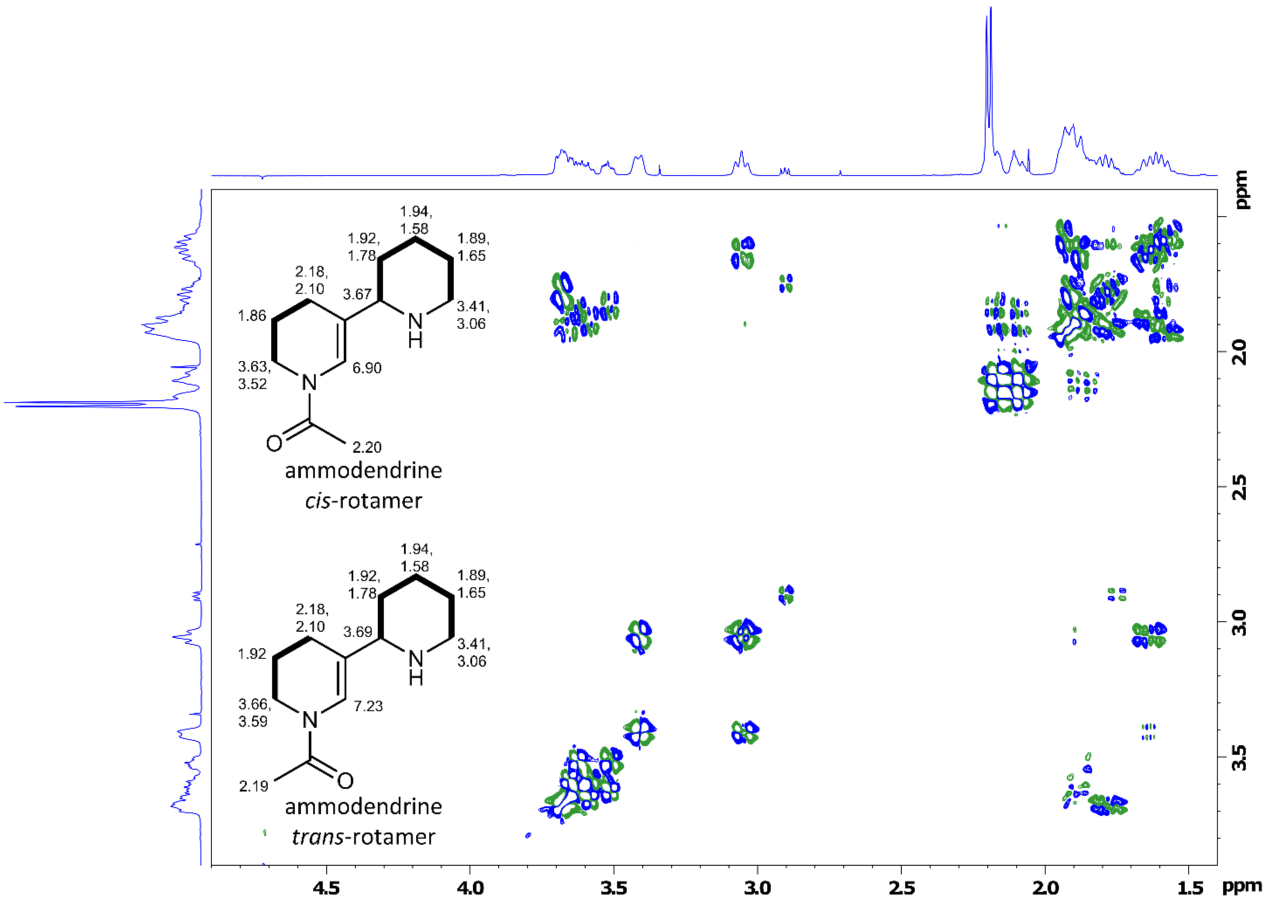

Fig. S5. DQF-COSY spectrum of ammodendrine (6) formate (600 MHz, D_2O, 300 K, DSS). Bold bonds indicate the main vicinal correlations for both rotamers. Proton resonances are added to aid interpretation.

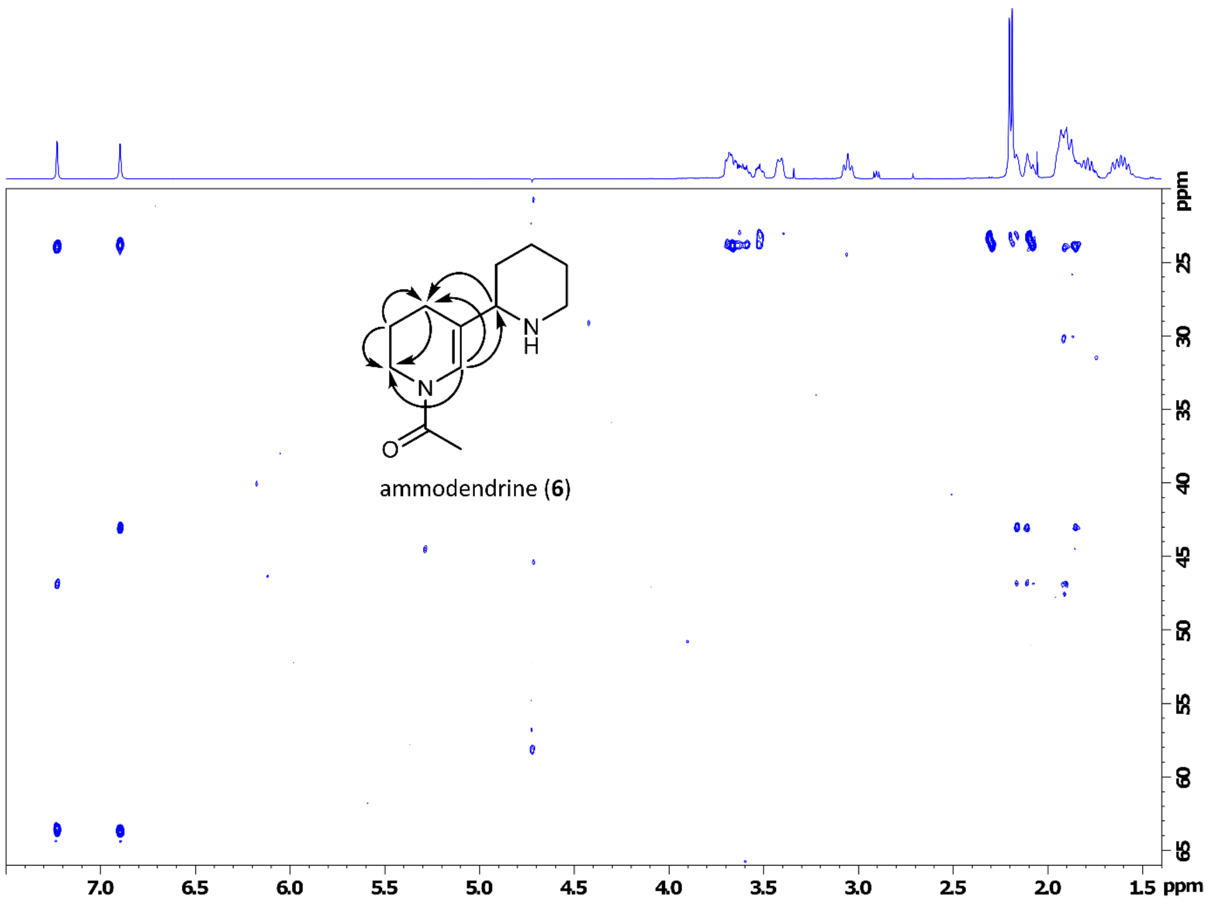

Fig. S6. ^{1}H-^{13}C-HMBC spectrum of ammodendrine (6) formate (600 MHz, D_2O, 300 K, DSS). Aliphatic carbon region. Arrows indicate key correlations for both rotamers.

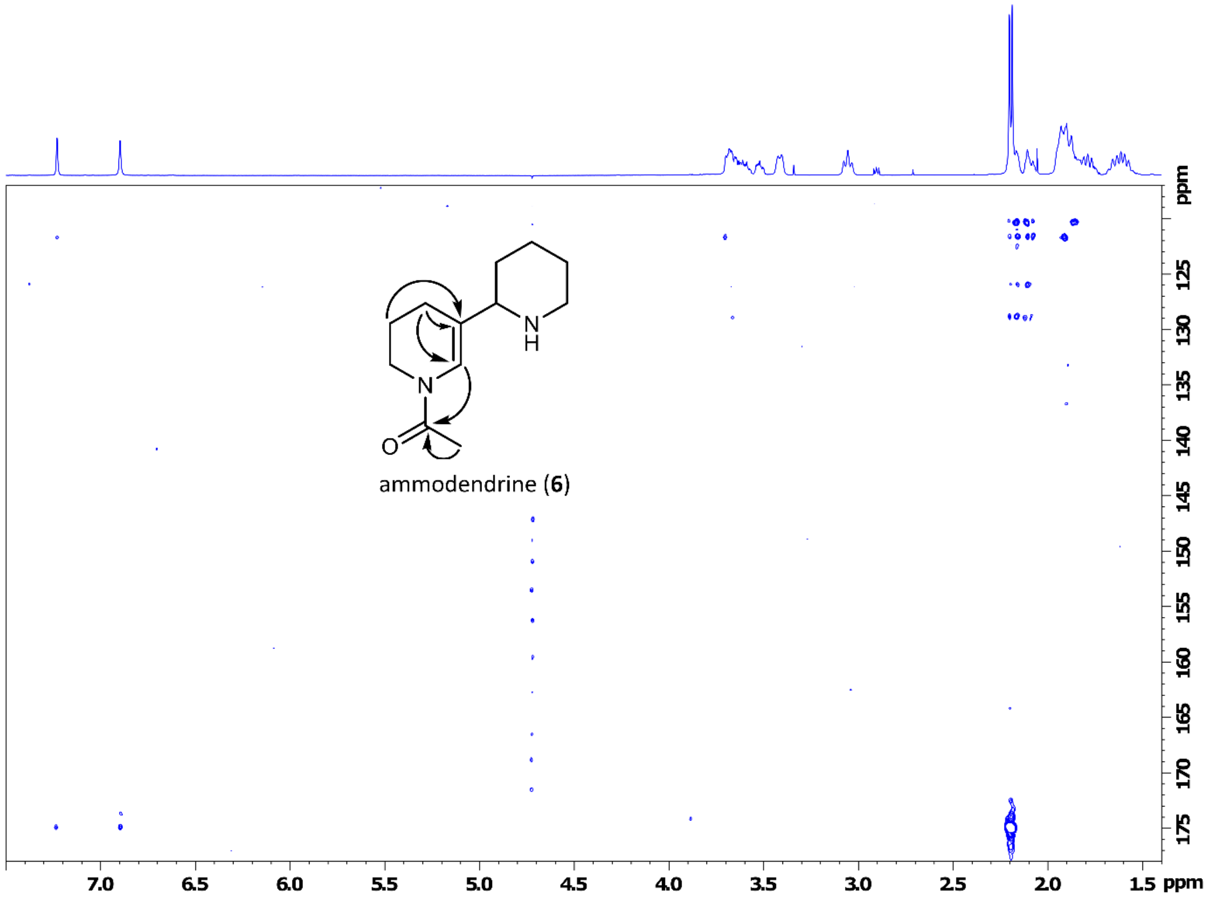

Fig. S7. ^{1}H-^{13}C-HMBC spectrum of ammodendrine (6) formate (600 MHz, D_2O, 300 K, DSS). Olefinic and carbonylic carbon region. Arrows indicate key correlations for both rotamers.

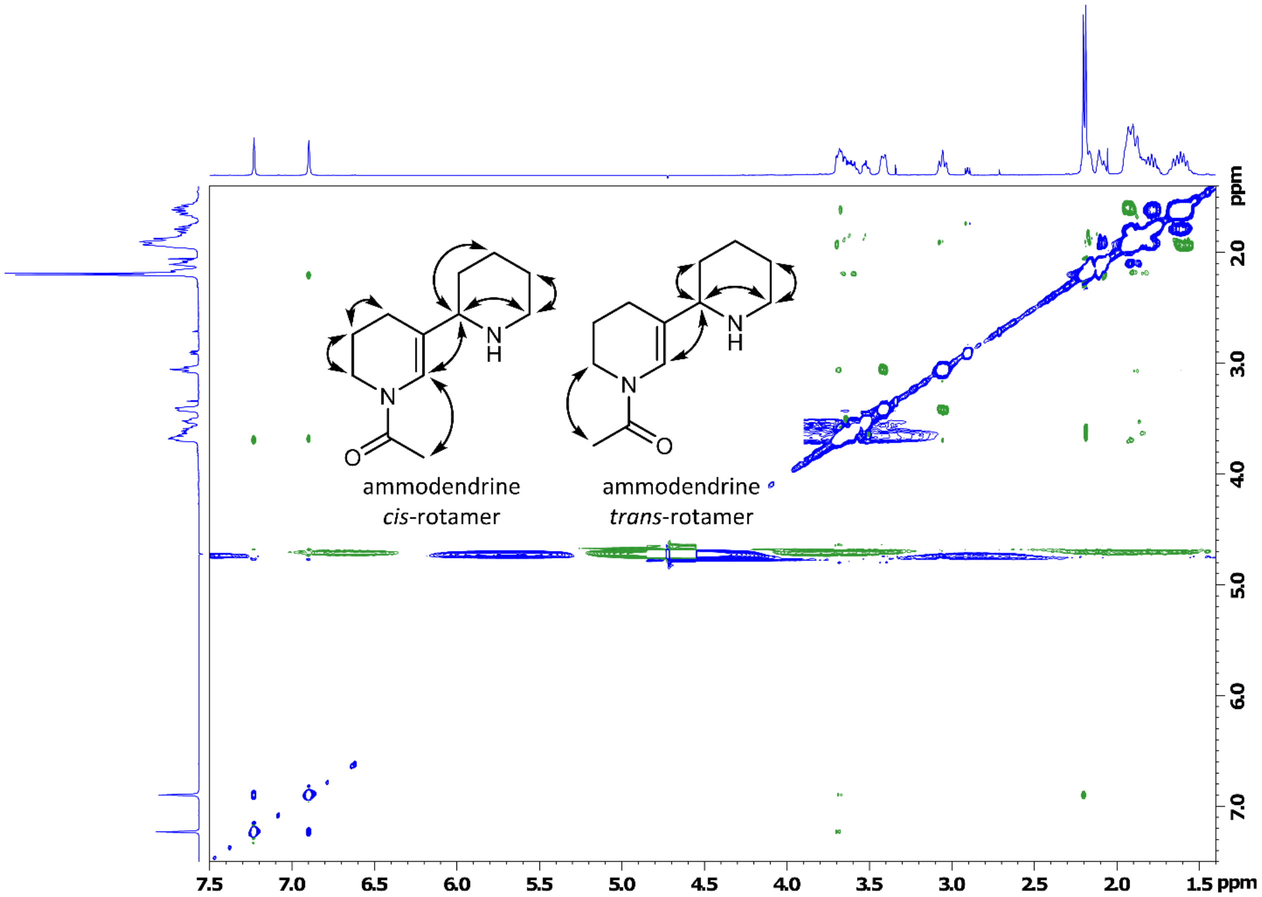

Fig. S8. ROESY spectrum of ammodendrine (6) formate (600 MHz, D_2O, 300 K, DSS). Arrows indicate key correlations due to rotating-frame Overhauser effects (ROEs).

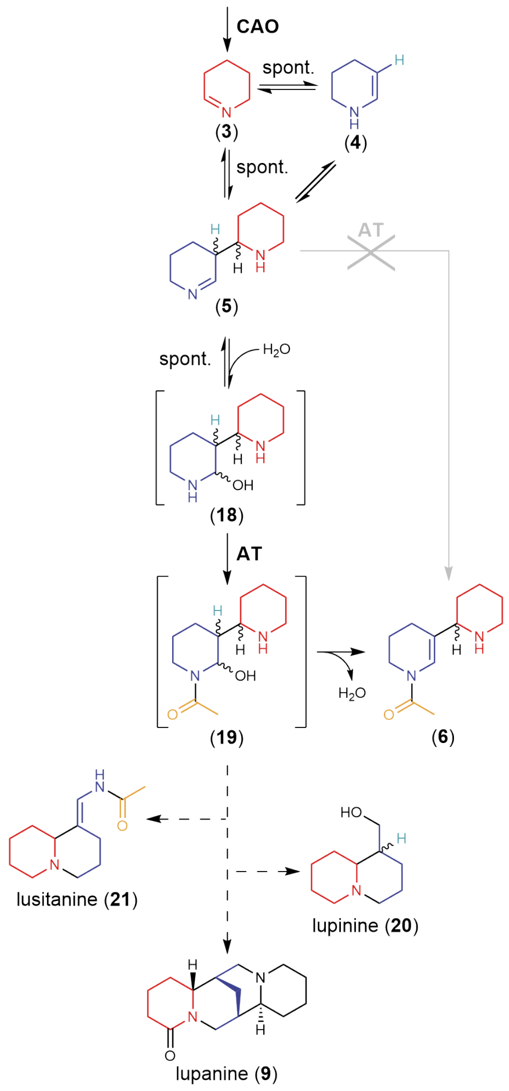

Fig. S9. Revised pathway hypothesis for the biosynthesis of QAs. We propose that acetylation by AT is a key step in the early biosynthesis of QAs (e.g., lupanine, **9**) and that the pathway proceeds through acetylated intermediates past the formation of the quinolizidine moiety, as suggested by the accumulation of the acetylated QA lusitatine (**21**) in several different lupin species (ref. 25 – Wink et al., 1995). Although tetrahydroanabasine (**5**) and ammodendrine (**6**) would appear to be the substrate and product of AT based on *in vitro* assays (light grey arrow), the imine nitrogen of **5** is only weakly nucleophilic, and **6** lacks a specific hydrogen atom (shown in cyan) that must be retained all the way from Δ^2-piperideine (**4**). This hydrogen atom has been shown to be incorporated into the QA lupinine (**20**) via labeled precursor feeding experiments (ref. 11 – Mancinotti et al., 2022; ref. 34 – Robins and Sheldrake, 1994). Therefore, we propose that AT acts upon the hydrated form of **5**, the carbinolamine **18**, which likely exists in equilibrium with **6** under physiological conditions. The immediate product would be the carbinolamide **19**, which retains said hydrogen atom. **19** can proceed towards quinolizidine ring formation or alternatively dehydrate to the more stable ammodendrine (**6**). Note that our *in vitro* results confirm that the product of CAO, Δ^1-piperideine (**3**), can dimerize spontaneously at physiological pH (Fig. 3B); however, we cannot exclude that an enzyme catalyzes this dimerization *in vivo*. Hypothetical intermediates are surrounded by square brackets. Dashed arrows indicate multiple unknown steps.

Other Supplementary Materials for this manuscript include the following:

Table S1. Genotyping and phenotyping of a white lupin diversity panel comprising 150 individuals.

Table S2. Genotyping and phenotyping of the expanded white lupin diversity panel comprising 227 lines.

Table S3. Analysis of bitter white lupin accessions previously found to carry the Amiga SNP_2 variant.

Table S4. Total alkaloid content in the seeds of AT mutant narrow-leafed lupin plants.

Table S5. List of DNA oligos used in this study.
